# Evolution of replicative DNA polymerases in archaea and their contributions to the eukaryotic replication machinery

**DOI:** 10.3389/fmicb.2014.00354

**Published:** 2014-07-21

**Authors:** Kira S. Makarova, Mart Krupovic, Eugene V. Koonin

**Affiliations:** ^1^National Center for Biotechnology Information, National Library of Medicine, National Institutes of HealthBethesda, MD, USA; ^2^Unité Biologie Moléculaire du Gène chez les Extrêmophiles, Institut PasteurParis, France

**Keywords:** DNA replication, archaea, mobile genetic elements, DNA polymerases, enzyme inactivation

## Abstract

The elaborate eukaryotic DNA replication machinery evolved from the archaeal ancestors that themselves show considerable complexity. Here we discuss the comparative genomic and phylogenetic analysis of the core replication enzymes, the DNA polymerases, in archaea and their relationships with the eukaryotic polymerases. In archaea, there are three groups of family B DNA polymerases, historically known as PolB1, PolB2 and PolB3. All three groups appear to descend from the last common ancestors of the extant archaea but their subsequent evolutionary trajectories seem to have been widely different. Although PolB3 is present in all archaea, with the exception of Thaumarchaeota, and appears to be directly involved in lagging strand replication, the evolution of this gene does not follow the archaeal phylogeny, conceivably due to multiple horizontal transfers and/or dramatic differences in evolutionary rates. In contrast, PolB1 is missing in Euryarchaeota but otherwise seems to have evolved vertically. The third archaeal group of family B polymerases, PolB2, includes primarily proteins in which the catalytic centers of the polymerase and exonuclease domains are disrupted and accordingly the enzymes appear to be inactivated. The members of the PolB2 group are scattered across archaea and might be involved in repair or regulation of replication along with inactivated members of the RadA family ATPases and an additional, uncharacterized protein that are encoded within the same predicted operon. In addition to the family B polymerases, all archaea, with the exception of the Crenarchaeota, encode enzymes of a distinct family D the origin of which is unclear. We examine multiple considerations that appear compatible with the possibility that family D polymerases are highly derived homologs of family B. The eukaryotic DNA polymerases show a highly complex relationship with their archaeal ancestors including contributions of proteins and domains from both the family B and the family D archaeal polymerases.

## Introduction

Recent experimental and comparative genomic studies on DNA replication systems have revealed their remarkable plasticity in each of the three domains of cellular life (Li et al., 2013; Makarova and Koonin, 2013; Raymann et al., 2014). In particular, archaea, members of the prokaryotic domain that gave rise to the information processing systems of eukaryotes, show remarkable diversity even with respect to the core components of the replication machinery, the DNA polymerases (DNAPs) (Makarova and Koonin, 2013). The main replicative polymerases of archaea belong to the B family of Palm domain DNAPs (Burgers et al., 2001) which is also widely represented in eukaryotes, eukaryotic and bacterial viruses, as well as some bacteria; however, in bacteria, these polymerases appear to be of viral origin and are involved mainly in repair whereas replication relies on a distinct, unrelated enzyme (Gawel et al., 2008). In addition to the polymerase core, which consists of three domains known as palm, fingers and thumb, most of the B family DNAPs contain an N-terminal 3′-5′ exonuclease domain and a uracil-recognition domain (Hopfner et al., 1999; Steitz and Yin, 2004; Rothwell and Waksman, 2005; Delagoutte, 2012).

Family B DNAPs are present in all archaeal lineages, and many archaea have multiple paralogs some of which appear to be inactivated; at least two paralogs can be traced to the Last Archaeal Common Ancestor (LACA) (Rogozin et al., 2008; Makarova and Koonin, 2013). In addition to the archaeal chromosomes, family B DNAPs are encoded by several mobile genetic elements (MGEs) that replicate in archaeal cells and could contribute to horizontal transfer of DNAPs (Filee et al., 2002). In particular, family B DNAPs closely related to those found in the host species are encoded by haloarchaeal head-tailed viruses such as *Halorubrum* myoviruses HF1, HF2 (Filee et al., 2002; Tang et al., 2002) and HSTV-2 (Pietila et al., 2013) whereas more diverged protein-primed Family B DNAPs have been identified in other haloviruses such as His1 and His2 (Bath et al., 2006). Furthermore, recently, family B DNAPs have been identified in a new group of self-synthesizing mobile elements, called casposons because they apparently employ Cas1, originally known as a component of the CRISPR-Cas immunity systems, as their integrase (Makarova et al., 2013; Krupovic et al., 2014a).

In addition to the family B polymerases, most of the archaeal lineages, with the exception of the Crenarchaeota, encode the unique family D DNAP (Cann et al., 1998) that accordingly can be inferred to have been present in LACA. The family D polymerases consist of two subunits. The large subunit DP2 is a multidomain protein which forms a homodimer that is responsible for the polymerase activity (Shen et al., 2001; Matsui et al., 2011). The DP2 protein does not show significant sequence similarity with any proteins except for the two C-terminal Zn finger domains. The structure of the complete DP2 protein so far has not been solved but the structure of the N-terminal domain reveals a unique fold (Matsui et al., 2011). The small subunit DP1 contains at least two domains, an ssDNA-binding OB-fold, and a 3′-5′ exonuclease domain of the metallophosphatase MPP family. The DP1 protein is the ancestor of the small B subunits of eukaryotic replicative DNAPs that, however, have lost the catalytic amino acid residues of the 3′-5′ exonuclease (Aravind and Koonin, 1998; Klinge et al., 2009). Evidence has been presented that in euryarchaea the family D DNAP specializes in the synthesis of the lagging strand whereas the family B DNAP, PolB3, is involved in the leading strand synthesis (Henneke et al., 2005). However, at least in *Thermococcus kodakarensis*, the family D DNAP is sufficient for the replication of both strands (Cubonova et al., 2013). The Crenarchaeota lack the family D DNAP but possess at least one additional active DNAP of the B family, suggesting that the two distinct B family DNAPs specialize in the leading and lagging strand replication, respectively, as is the case in eukaryotes. In particular, biochemical data suggest that in *Sulfolobus solfataricus*, one family B polymerase (PolB1/Dpo1) is responsible for the synthesis of the leading strand whereas the other one, PolB3/Dpo3, is involved in the synthesis of the lagging strand (Bauer et al., 2012).

Some crenarchaeal and euryarchaeal plasmids encode palm domain polymerases of the archaeo-eukaryotic primase superfamily (Iyer et al., 2005), known as prim-pol, but in these plasmids the protein apparently is employed for initiation of replication rather than elongation (Iyer et al., 2005; Lipps, 2011; Krupovic et al., 2013; Gill et al., 2014).

Here we summarize the results of an updated comparative genomic and phylogenetic analysis of archaeal polymerases, focusing primarily on the diversity of Family B, including the polymerases associated with proviruses and mobile elements, and discuss their evolutionary relationships with eukaryotic DNAPs.

## Comparative genomic and phylogenetic analysis of archaeal DNA polymerases

### Phylogeny, domain architecture and gene neighborhoods of B family DNAPs in archaea

**Figure 1:**
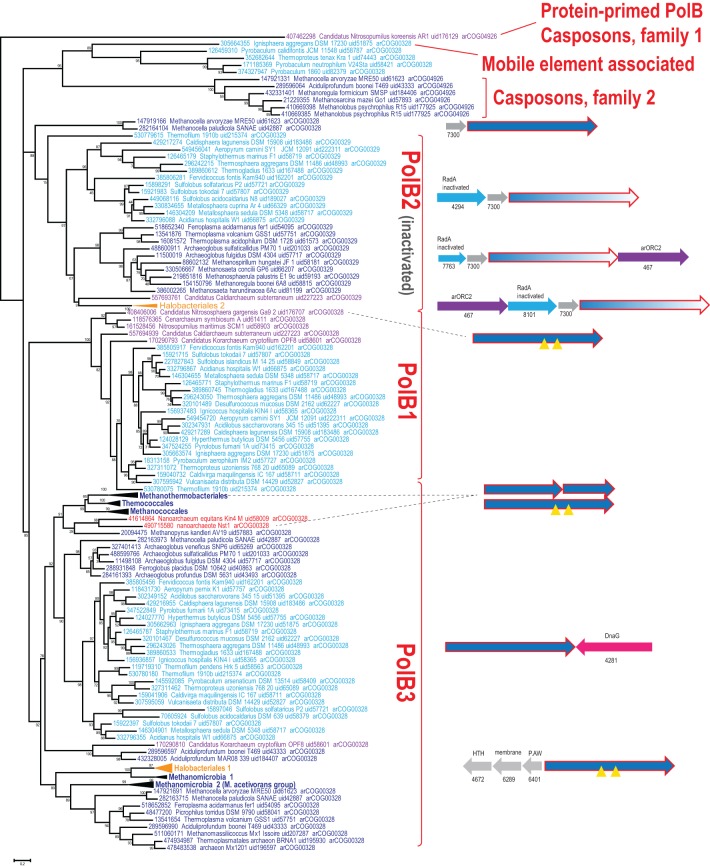
**Phylogenetic analysis of the polymerase B family in archaea**. The MUSCLE program (Edgar, 2004) was used for construction of sequence alignments. The tree was reconstructed using the FastTree program (Price et al., 2010) (179 sequences and 209 aligned positions). The complete tree is available in the Supplementary Figure S3. The sequences are denoted by their GI numbers, species names, refseq genome UID number and the arCOG number to which the respective protein currently assigned. Several branches are collapsed and shown as triangles denoted by the respective lineage taxonomy name. Color code: Euryarchaeota, dark blue, with the exception of Halobacteria that are shown in orange; Crenarchaeota, light blue; deeply branched archaeal lineages (Thaumarchaeota, Korarchaeota, Nanoarchaeota), purple; Nanoarchaea, red. The conserved neighborhoods (if any) are shown on the right side of the tree for the respective branches. Homologous genes are shown by arrows of the same color; genes are shown approximately to scale. Color code: polymerase genes are shown by red outline, inteins are shown by yellow triangles, uncharacterized genes are rendered in gray. The arCOG numbers are provided underneath the respective gene arrows for all non-polymerase genes. Abbreviations: arORC2—ORC/CDC6 AAA+ ATPases, arORC2 subfamily (Makarova and Koonin, 2013), HTH—helix-turn-helix; P.AW—the conserved motif for the respective uncharacterized protein.

**Figure 2:**
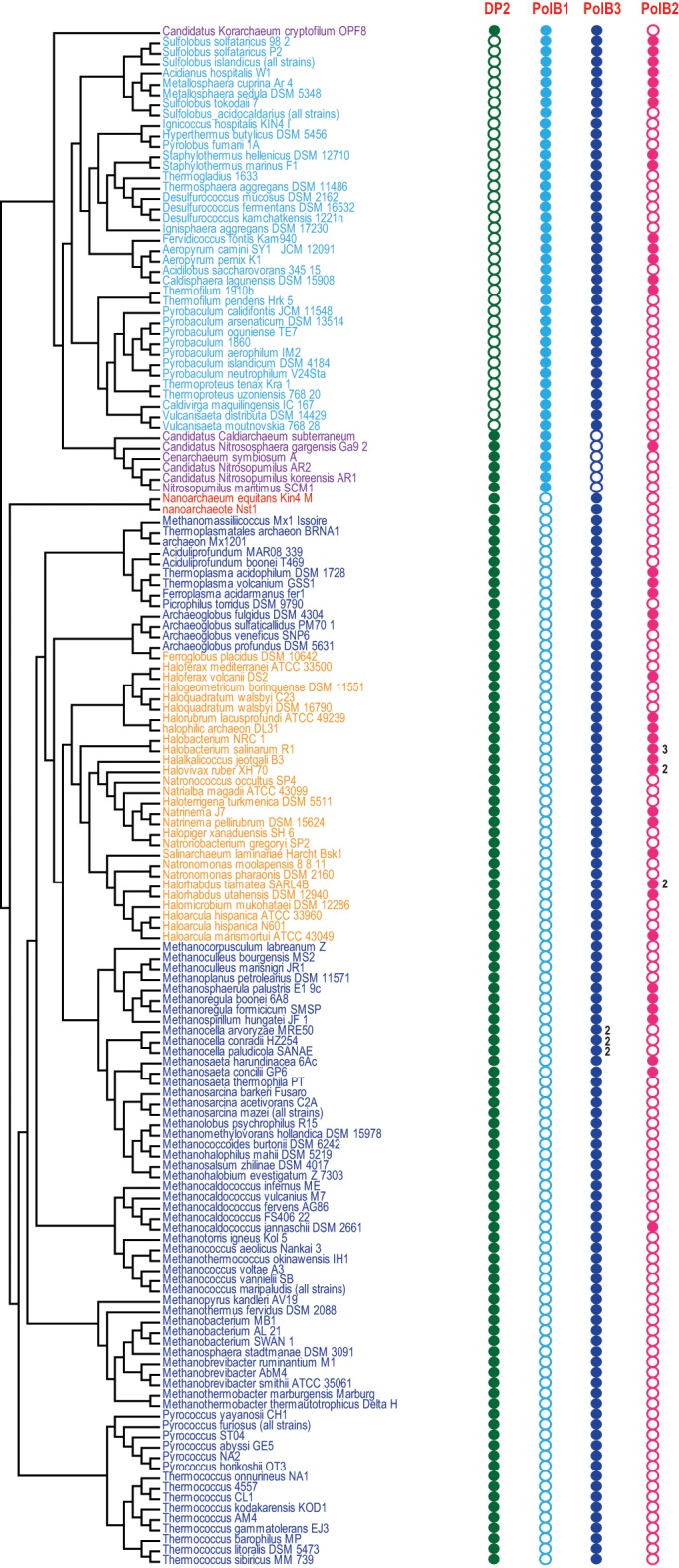
**Phyletic patterns for the major groups of archaeal DNAPs**. Phyletic patterns for the indicated protein families (filled circles show presence and empty circles show absence) are superimposed over the phylogenetic tree of archaea. The number of paralogs are indicated by a number, if more than one paralog of the respective family is encoded in the genome. The tree topology is based on the phylogeny of concatenated ribosomal proteins.

Using the latest recent update of archaeal clusters of orthologous genes (arCOGs) (Wolf et al., 2012) which includes 168 complete genome sequences of archaea (Refseq update as of February 2014), we reconstructed a phylogenetic tree of family B polymerases for a representative set of archaeal genomes and analyzed their gene context (Figure 1). One of the selected sequences (YP_006773615 from *Candidatus* Nitrosopumilus koreensis) belongs to the distinct, protein-primed DNAP family (see discussion below) and thus was used as an outgroup (Figure 1). Another protein (YP_007906966 from *Archaeoglobus sulfaticallidus*) is extremely diverged and poorly alignable and therefore has not been included in the tree reconstruction. Consistent with previous observations (Edgell et al., 1998; Rogozin et al., 2008), the tree encompassed three large branches: (i) PolB3, the “major” DNAP, present in all archaea except Thaumarchaeota, (ii) PolB1, the “minor” DNAP, present only in the TACK (Thaumarchaota, Aigarchaota, Crenarchaeota and Korarchaeota) superphylum (Guy and Ettema, 2011; Martijn and Ettema, 2013) and (iii) PolB2, a distinct family of DNAP homologs most of which appear to be inactivated as inferred from the replacement of the catalytic amino acid residues (Rogozin et al., 2008) and show a patchy distribution in most archaeal lineages (Figures 1, 2, Supplementary Table S1).

Despite the presence in most archaeal genomes, the PolB3 branch shows little topological congruence with the archaeal phylogeny that was established primarily through phylogenetic analysis of multiple translation, transcription and replication system components (Guy and Ettema, 2011; Yutin et al., 2012; Podar et al., 2013; Raymann et al., 2014). The deviations include the polyphyly of Euryarchaeota, Methanomicrobia, and Thermoplasmatales, and paraphyly of Sulfolobales-Desulfurococcales with respect to Thermoproteales. These discrepancies suggest that the history of archaeal Family B DNAPs included multiple horizontal gene transfer (HGT) events and/or major accelerations of evolution. No recent duplications are observed within this group of polymerases but some archaea possess two versions of PolB3 that could have different origins. In particular, acquisition of two versions of PolB3 (one from Archaeoglobales and another from Thermoplasmatales), followed by the loss of the ancestral methanomicrobial gene, seems likely for the genus *Methanocella*.

Several groups of archaea contain intein insertions in the PolB3 gene, up to three per gene (Perler, 2002). Inteins are parasitic genetic elements that insert into protein-coding genes, perform self-splicing at the protein level and typically encode an endonuclease that mediates intein gene propagation into ectopic DNA sites (Perler et al., 1994; Gogarten et al., 2002). The majority of intein insertion sites in PolB3 genes are shared between different archaea but some are lineage-specific (Perler, 2002; MacNeill, 2009). It appears likely that the split PolB3 genes in Methanobacteriales (Kelman et al., 1999) evolved as a result of erratic intein excision, especially considering that in the tree these split DNAP genes cluster with Methanococcales and Thermococcales which both contain inteins in PolB3 genes (Figure 1). Similarly, a split PolB gene, in this case with the two parts non-adjacent, is found in *Nanoarchaeum equitans* where it could be trans-spliced via an intein parts of which are associated with the two split gene fragments (Perler, 2002; Choi et al., 2006). In the recently sequenced nanoarchaeon Nst1, the orthologous PolB3 gene is not split (Podar et al., 2013), suggesting that intein insertion and split occurred late in the evolution of the Nanoarchaeota.

In most of the archaea, PolB3 genes do not form conserved genomic neighborhoods. The only notable exception is a conserved genomic context of this gene in most crenarchaea that includes the bacterial-type DNA primase *dnaG*; however, the *polB3* and *dnaG* genes are oriented convergently and accordingly are transcribed from different promoters. In addition, in all haloarchaeal genomes, PolB3 might be co-regulated with three uncharacterized genes that are specific to this group of archaea (e.g., HVO_0855-HVO_0857 from *Haloferax volcanii*); the protein product of one of these genes (HVO_0855) contains a helix-turn-helix DNA-binding domain, suggesting that it could be a regulator of PolB3 transcription (Figure 1).

The second major branch of archaeal family B DNAPs includes the replicative polymerases of the PolB1 group that is represented in all members of the TACK superphylum (Figure 2). Most of the Thaumarchaeota possess only this form of active family B DNAP whereas Korarchaeaum and Crenarchaeota encode both PolB3 and PolB1. In a striking contrast to PolB3, the topology of this branch is almost fully consistent with the archaeal phylogeny, indicative of a primarily vertical mode of evolution of this gene. So far only in *Nitrososphaera gargensis*, two inteins are inserted into the PolB1 gene (Figure 1).

The third large group of family B DNAPs that includes the experimentally characterized PolB2/Dpo2 of *S. solfataricus* shows a patchy distribution in archaea but is rapidly growing with the sequencing of new genomes that have been found to encompass this gene, along with several bacteria (Rogozin et al., 2008). The PolB2 family is currently represented in Crenarchaeota, Euryarchaea and also in *Caldiarchaeum subterraneum*, the only known member of the putative phylum Aigarchaeaota (Figure 1). The topology of this branch is generally consistent with a predominantly vertical mode of evolution, along with multiple losses in several archaeal lineages. It appears likely that in the case of this group, the deviations from the archaeal phylogeny are due primarily to increased rates of evolution of this gene in some lineages (Figure 1). Thus, along with the PolB3 and PolB1 groups, PolB2 probably was already represented in LACA. Sequence comparison of this subfamily with other family B DNAPs shows that, in most members, multiple catalytic residues of both the polymerase and the exonuclease domains are replaced, suggesting that these proteins are inactivated DNAPs (Rogozin et al., 2008). However, very weak activities of both enzymatic domains have been reported for a single member of this group, PolB2/Dpo2 of *S. solfataricus* (Choi et al., 2011).

Recent comparative genomic analysis identified an association between PolB2 genes, an uncharacterized gene of arCOG07300 and a *radA*-like gene in Sulfolobales (Makarova and Koonin, 2013). We analyzed the genomic neighborhoods for this family in greater detail and found that many diverged members of arCOG07300 have been missed originally due to the low sequence similarity with proteins from Sulfolobales but were now detected by using more sensitive methods, such as PSIBLAST, allowing to expand the family considerably (Supplementary Figure S1). The arCOG07300 proteins are small (~90 aa), alpha-helical proteins that do not show statistically significant similarity with any available protein sequences. Three arCOGs (arCOG07763, arCOG04294, arCOG08101) in the predicted operons with the inactivated polymerase PolB2 and arCOG07300 belong to the RadA family but all appear to be inactivated as judged by the substitution of the key amino acid residues implicated in ATP binding and hydrolysis (Supplementary Figure S2). In one of these proteins (arCOG07763), the P-loop ATPase domains deteriorated so severely that similarity to RadA could be detected only using such sensitive methods as HHpred (Supplementary Figure S2). Because the phyletic patterns of arCOG07763, arCOG04294, arCOG08101 are complementary and the respective genes are embedded in the same genomic context, these genes appear to be orthologs that have evolved at high rates, losing readily detectable sequence similarity. Several haloarchaea possess an additional copy of a two gene operon that consists of arCOG07763 and arCOG07300. In two *Methanocella* species, the arCOG07300 gene is also present in the same operon with predicted active DNAPs which form the sister group to the inactivated PolB2/Dpo2 (Figure 1), suggesting that the functional link with arCOG07300 evolved before polymerase inactivation. In many euryarchaeal genomes, the neighborhood also includes an arORC2 family gene (Makarova and Koonin, 2013), an ATPase component of origin recognition complex (Figure 1). The same three gene families are linked also in the several bacterial genomes that encode a PolB2 homolog. In addition to these three genes, in some bacteria, *lexA*, the SOS response master repressor gene, is located in the same predicted operon. This association implies that the putative protein complex encoded by this operon is involved in DNA damage response. A typical example of these associations is a locus in *Leptospirillum ferriphilum* that consists of four genes LFML04_0990-LFML04_0993 encoding, respectively, an “inactivated” polymerase, a homolog of arCOG07300, inactivated *radA* and *lexA*. Taken together, these observations indicate that PolB2/Dpo2, inactivated RadA and arCOG07300 proteins are most likely functionally linked and could also form a complex given that proteins encoded in evolutionarily conserved operons often interact both physically and functionally (Dandekar et al., 1998; Quax et al., 2013).

Given its wide spread and likely ancestral provenance in archaea, this complex might perform important, albeit dispensable function in DNA damage repair, more specifically, perhaps in the elimination of stalled replication forks, and/or in the regulation of DNA replication.

The presence of the arORC2 family gene, which encodes an ATPase component of the origin recognition complex, in the same neighborhood of many euryarchaeal genomes implies a replication-related function (Makarova and Koonin, 2013) (Figure 1). Recently, it has been shown that in *Haloferax volcanii* a RadA protein is required for initiation of replication in origin-less cells (Hawkins et al., 2013). Although the RadA shown to be involved in this process is an active ATPase and belongs to a different family (arCOG00417), given the association of the putative PolB2-inactive RadA-arCOG07300 operon with the arORC2 gene, the complex of these proteins might be involved in an alternative mechanism of replication initiation or in the regulation of origin recognition. Clearly, an important aspect of the further characterization of this predicted complex is the determination of the presence or absence (as suggested by comparative sequence analysis) of enzymatic activities in PolB2.

Several archaea possess another, divergent B family DNAP (arCOG04926) that is predicted to be active. Recently, it has been shown that this gene is tightly associated with several other genes, including Cas1 (a CRISPR-Cas system gene), and belongs to a new class of mobile elements called Casposons (see details below). A sister branch of this family includes active polymerases from several closely related genomes of Thermoproteales and a single representative of Desulfurococcales, *Ignisphaera aggregans* (Figure 1). In *I. aggregans,* the DNAP gene of this group probably belongs to a provirus (see below) whereas the respective genes in Thermoproteales do not display any conserved genomic associations and are unlikely to belong to mobile genetic elements although their origin from such elements cannot be ruled out.

### DNA polymerases encoded within integrated mobile elements

Mobile genetic elements (MGE), such as viruses and plasmids, often encode their own genome replication proteins. In archaea, viruses from at least four different families are known to encode DNA polymerases. Tailed viruses of the order *Caudovirales* encode RNA-primed family B DNA polymerases (PolB) (Sencilo et al., 2013), whereas certain members of the families *Ampullaviridae* (Peng et al., 2007), *Fuselloviridae* (Bath et al., 2006; Krupovic et al., 2014b) and *Pleolipoviridae* (Bath et al., 2006; Pietila et al., 2012) carry genes for protein-primed PolBs. Integration of MGEs that contain genes for cellular-like replication proteins into the host chromosome can be and often is confused with the duplication of the *bona fide* cellular genes encoding these proteins (Krupovic et al., 2010; Forterre and Prangishvili, 2013). Therefore, careful gene neighborhood analysis is necessary to ascertain the provenance of replication protein genes in genomes of cellular organisms, especially when multiple paralogs of a given gene appear to be present.

**Figure 3:**
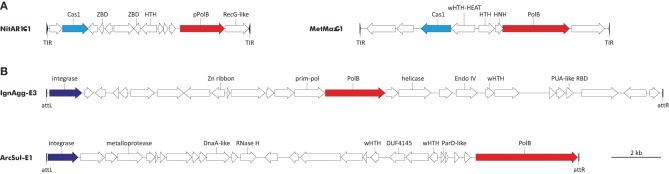
**Genome maps of archaeal PolB-encoding mobile genetic elements. (A)** Casposons of families 1 and 2. NitAR1-C1 is present in the genome of *Candidatus Nitrosopumilus koreensis* AR1 (NC_018655; nucleotide coordinates: 655308 to 663492), whereas MetMaz-C1 is from *Methanosarcina mazei* Go1 (NC_003901; nucleotide coordinates: 3946601 to 3956653). **(B)** Tyrosine recombinases-encoding elements. IgnAgg-E3 is found in the genome of *Ignisphaera aggregans* DSM 17230 (NC_014471; nucleotide coordinates: 1844012 to 1868704) and ArcSul-E1 is from *Archaeoglobus sulfaticallidus* PM70-1 (NC_021169; nucleotide coordinates: 873590 to 894826). Predicted protein-coding genes are indicated with arrows, indicating the direction of transcription. Genes for PolBs are shown in red, *cas1* genes are in cyan, and genes for tyrosine recombinases are colored blue. Abbreviations: TIR, terminal inverted repeats; att, attachment site; ZBD, Zinc-binding domain-containing protein; HNH, HNH family endonuclease; (w)HTH, (winged) helix-turn-helix proteins; RBD, RNA-binding domain.

With regard to DNAPs, two types of elements encoding diverse family B polymerases are integrated in the genomes of diverse archaea (Figure 3). The first group includes the recently discovered transposon-like elements called Casposons (Krupovic et al., 2014a). Unlike other known mobile genetic elements, casposons apparently rely on Cas1 endonucleases, the key enzymes of the prokaryotic CRISPR-Cas immunity (hence the name), for integration into the cellular genome. These elements are found in both bacteria and archaea. Casposons are 7–20 kb in length and are surrounded by terminal inverted repeats and target site duplications (Figure 3). Three families of casposons have been defined based on the phylogenetic analysis of the Cas1 endonucleases, gene content and taxonomic distribution. Family 1 casposons are thus far exclusively found in Thaumarchaeota (4 elements) and encode protein-primed PolBs that are most closely related to the corresponding proteins of archaeal viruses His1 (*Fuselloviridae*) and His2 (*Pleolipoviridae*). Phylogenetic analysis of the viral and casposon pPolB suggests that there has been exchange of the pPolB genes between these two types of MGEs (Krupovic et al., 2014a).

Casposons of families 2 and 3 encode typical RNA-primed PolBs and are respectively found in the genomes of euryarchaeota (11 casposons) and bacteria (4 casposons). In the phylogenetic tree of PolB, these bacterial and archaeal casposons form a clade that emerges as a sister group to the DNAPs of different species of the crenarchaeal class Thermoprotei (Figure 1). Notably, in the latter group, PolB of *Ignisphaera aggregans* DSM 17230 is also encoded within an integrated mobile element which is, however, unrelated to the casposons (see below).

The second type of PolB-encoding MGEs includes two elements, IgnAgg-E3 (24.7 kb) and ArcSul-E1 (21.2 kb), found in the genomes of the crenarchaeon *I. aggregans* and the euryarchaeon *Archaeoglobus sulfaticallidus* PM70-1, respectively (Figure 3). These two elements share genes neither with each other nor with known archaeal viruses or plasmids (a detailed description of IgnAgg-E3 and ArcSul-E1 will be published elsewhere) and accordingly could be founding members of two new groups of MGEs.

### Evolutionary relationships of archaeal and eukaryotic DNA polymerases

Based on the above considerations and the respective phyletic patterns, three family B polymerases, PolB1, PolB2 (“inactivated” DNAPs) and PolB3, could be projected to LACA. In addition to these three families, two subunits of family D polymerase, arCOG04455 and arCOG04447, and a family Y polymerase, arCOG04582 (see the respective phyletic patterns in the Supplementary Table S1) also are likely to be ancestral. A polymerase of family X, although common in archaea, cannot be projected to LACA with confidence (Wolf et al., 2012). The latter two polymerases (families X and Y) are unlikely to be involved in genome replication. In bacteria and eukaryotes, members of both families have been thoroughly characterized and shown to function in DNA repair (Jarosz et al., 2007; Moon et al., 2007; Silverstein et al., 2010; Sharma et al., 2013). The experimental data suggests that PolB1 and the family D DNAP are the main replicative polymerases in crenarchaea and euryarchaea, respectively, whereas PolB3 appears to be involved in the replication of the lagging strand in most archaea (Cubonova et al., 2013).

Most eukaryotes possess four paralogous family B DNAPs denoted Pol-α, Pol-δ, Pol-ε, and Pol-ζ, four family Y polymerases (Yang, 2014), four family X polymerases (Bebenek et al., 2014) and two family A polymerases involved in mitochondrial replication and DNA repair (Burgers et al., 2001). All these polymerases seem to have been present in the last eukaryotic common ancestor (LECA). The functions of family B polymerases in eukaryotes are diversified: Pol-ε is the main replicative polymerase specialized in the replication of the leading strand, Pol-δ replicates the lagging strand, Pol-α is the main component of the eukaryote-specific primase complex, which synthesizes short DNA primers during the lagging strand replication (Kunkel and Burgers, 2008; Pavlov and Shcherbakova, 2010 and references therein), and Pol-ζ is involved in lesion bypass (Sharma et al., 2013). Furthermore, the functions of all family B DNAPs in eukaryotes require an additional small subunit, the same for all family B DNAPs (Bell and Dutta, 2002).

**Figure 4:**
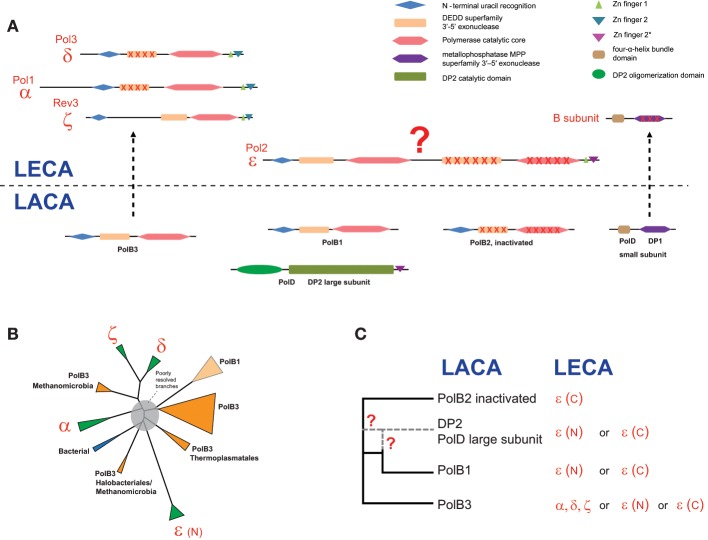
**Reconstruction of the complements of replicative DNAPs in the last archaeal and eukaryotic ancestors and a hypothetical scenario of their evolutionary relationships. (A)** Polymerase B and D family genes projected to archaeal (LACA) and eukaryotic (LECA) last common ancestors and their domain organization. Homologous domains are shown by shapes of the same color. Inactivated domains are crossed. For eukaryotic polymerase families, human and yeast gene names are provided. **(B)** The unrooted phylogenetic tree of active polymerases of B family. The MUSCLE program (Edgar, 2004) was used for construction of multiple sequence alignments. The tree was reconstructed using the FastTree program (Price et al., 2010) (141 sequences and 264 aligned positions). The complete tree is available in the Supplementary Figure S4. The tree is rendered as a scheme, with all major groups collapsed. **(C)** The inferred evolutionary relationships between archaeal and eukaryotic replicative DNAPs. Details on the involvement of PolD in the evolution of eukaryotic DNAPs are discussed in the text. The question mark denotes an uncertainty in evolutionary scenario.

Domain architectures and the relationships between archaeal and eukarytic replicative polymerase families are schematically shown in Figure 4A. The small subunits evolved from the small subunit (DP1) of the archaeal family D polymerase which in archaea is an 3′-5′-exonuclease of the MPP superfamily that appears to be involved in proofreading during archaeal DNA replication (Aravind and Koonin, 1998; Jokela et al., 2004). However, the homologous small subunit of the eukaryotic DNAPs has lost the catalytic amino acid residues and performs an architectural role in the DNAP complex (Aravind and Koonin, 1998; Klinge et al., 2009; Yamasaki et al., 2010).

The evolutionary relationships between the polymerase subunits themselves are much more difficult to establish due to the multiplicity of paralogs in both archaea and eukaryotes, and the apparent differences in the evolutionary rates resulting in poorly resolved phylogenetic tress (Edgell et al., 1998; Filee et al., 2002; Tahirov et al., 2009). Furthermore, due to the use of considerably different sets of sequences and different methods of tree reconstruction employed, the results of different analyses are not directly comparable. The only observation that seems to be fully consistent is the grouping of eukaryotic polymerases δ and ζ. We made another attempt to reconstruct a phylogenetic tree of the family B DNAPs including only major branches of active polymerases (hence excluding PolB2) from archaea, eukaryotes and bacteria, and using an updated, representative set of sequences (Figure 4B). In the resulting tree, most of the deep branches are poorly resolved and unstable depending on the set of sequences used and the method of tree reconstruction (data not shown). The only additional observation that appears reliable is the confident grouping of PolB3 from several Methanomicrobia with the eukaryotic branch containing DNAPs δ and ζ (Figure 4B); this affinity is supported by the relatively high BLAST scores of the pairwise alignments of these sequences to eukaryotic polymerases compared with other archaeal polymerases (Supplementary Table S2). However, the PolB3 sequences from these Methanomicrobia lack the two Zn fingers at the C-terminus, a synapomorphy of the eukaryotic family B DNAPs that is also present in the archaeal PolD (Tahirov et al., 2009) (Figures 1, 4A and see discussion below). If grouping of PolB3 from Methanomicrobia with DNAPs δ and ζ reflects an actual evolutionary event, then a complicated scenario would have to be proposed, including acquisition of a eukaryotic polymerase by the ancestor of this group of organisms, loss of the “original” PolB3 and loss of the C-terminal Zn-fingers in the acquired polymerase. The alternative is the even less plausible scenario whereby the common ancestor of the eukaryotic DNAPs δ and ζ evolved from an unknown variant of the methanomicrobial PolB3 that contained at least one Zn finger; however, this scenario contradicts the recent conclusions on the origin of eukaryotes from the archaeal TACK superphylum (Martijn and Ettema, 2013; Koonin and Yutin, 2014). Given the complexity of these scenarios, the possibility should be considered that, the apparent strong support notwithstanding, the eukaryote-methanomicrobial affinity is yet another tree reconstruction artifact caused by large differences in evolutionary rates in different branches.

Thus, we have to conclude that phylogenetic analysis fails to resolve the evolutionary relationships between archaeal and eukaryotic family B DNAPs. So could any other considerations help understanding the origin of family B DNAPs that are responsible for eukaryotic DNA replication? In particular, this puzzle cannot be solved in full without uncovering the provenance of the family D polymerases, especially taking into account that the DP1 subunit clearly made it to LECA and is an indispensable component of all replicative B-family polymerases in eukaryotes (Yamasaki et al., 2010) whereas the DP2 subunit appears to have been lost. Furthermore, there is a significant, specific sequence similarity between the C-terminal Zn fingers of Pol-ε and DP2 (Tahirov et al., 2009). Any scenarios that strive to accommodate all these findings require intricate chains of events (Tahirov et al., 2009).

An intriguing possibility is suggested by the conservation of several aspartate residues in the catalytic domain of DP2, including the DxD motif that is present in all palm domain polymerases and is involved in the binding of an essential divalent cation (Cann et al., 1998). This observation might indicate that, notwithstanding the absence of readily detectable sequence similarity, DP2 is a highly derived homolog of family B DNAPs. This hypothesis appears to be able to accommodate all available facts in the simplest possible fashion. The fact that the small subunit of the family D DNAP, DP1, is the readily detectable ortholog of the B subunit that is shared by all eukaryotic family B polymerases is also compatible with this scenario. It has been shown that the eukaryotic Pol-ε consists of an N-terminal DNAP domain in which all major catalytic motifs of family B are conserved and a C-terminal DNAP domain in which most of these motifs are disrupted suggestive of inactivation(Tahirov et al., 2009).The present hypothesis could account for the origin of Pol-ε as a fusion of an ancestral form of DP2 (before its accelerated evolution period) that would give rise to the active, N-terminal domain of Pol-ε, followed by an inactivated PolB2 domain inserting between the active N-terminal domain and the Zn finger (Figure 4). The N-terminal polymerase domain of Pol-ε shows a pattern of insertions and deletions that is distinct from those in all other family B DNAPs, which is compatible with a distinct origin (Tahirov et al., 2009). The accelerated evolution of the PolB1 at the origin of DP2 might have occurred within a viral genome, followed by reintroduction of this evolved gene into an ancestral euryarchaeal lineage via the so-called host-to-virus-to-host transfer loop, as has been proposed for the replicative MCM helicases of Methanococcales (Krupovic et al., 2010). In functional terms, this hypothesis is compatible with the fact that Pol-ε is the leading strand polymerase in eukaryotes. Obviously, this hypothesis will be put to test when the structure of the catalytic domain of DP2 is solved. Furthermore, the possibility remains that genome sequencing of currently uncharacterized, deep branches of archaea results in identification of novel DNAPs that help clarifying the relationships between the B and D families, and possibly, other aspects of DNAP evolution.

## Conclusions

The DNAPs comprise the core of the DNA replication machinery, obviously one of the key functions in any cellular life form and many viruses and mobile elements. Other genes involved in key information processing functions, such as the core components of the translation and transcription systems, have highly conserved sequences, are rarely duplicated and do not seem to experience major accelerations of evolution (Koonin, 2003; Puigbo et al., 2009). Therefore, reconstruction of the evolution of the respective systems is a relatively straightforward task. Their major biological importance notwithstanding, the DNAPs evolve under a different regime that appears to involve multiple duplications, gene losses, horizontal gene transfers and domain rearrangements. Moreover, inactivated DNAPs seem to have adopted new functions the exact nature of which remains to be elucidated. The complexity of the evolution of the DNAPs is likely to stem partly from the functional differentiation because in archaea and eukaryotes the lagging and leading strand are replicated by distinct DNAPs. Another important factor is the common presence of DNAPs in viruses and other mobile genetic elements that can transfer the DNAP genes between cellular organisms, providing an environment conducive to accelerated evolution, and possibly replacing the original genes.

The starkest manifestation of the complexity of DNAP evolution is the intricate relationship between the archaeal and eukaryotic replication machineries. Here we proposed a parsimonious evolutionary scenario under which the archaeal family D of DNAPs is a highly derived form of family B. However, the available data are also compatible with various other scenarios that would involve contributions from different archaeal DNAPs and possibly also viruses.

### Conflict of interest statement

The authors declare that the research was conducted in the absence of any commercial or financial relationships that could be construed as a potential conflict of interest.
