# Supplementary material for: Evolution of replicative DNA polymerases in archaea and their contributions to the eukaryotic replication machinery
Source: Front Microbiol. 2014 Jul 21;5:354. doi: 10.3389/fmicb.2014.00354 (PMC4104785; doi:10.3389/fmicb.2014.00354)
Supplement: Supplementary file 1 [file DataSheet1.PDF]

Supplementary Figure S1. Multiple alignment of arCOG07300 protein family

Multiple alignment of the arCOG07300 family was built using Muscle program.
Secondary structure was predicted using Jpred program and is shown above the alignment (H - alpha helix). The sequences are denoted by Genbank identifier and full organism name.

Archaea

```
Secondary structure (JPRED)                                 HHHHHHHHHHHH    HHHH H    HHHHHHHHHHHHHHHHH            HHHHHHHHHHHHHHHH
449068115 Sulfolobus_acidocaldarius_N8              MGRTQPSFTRAVDAELAKLL----RLSE-RIGYPCFREVIVEATKRVRDFQSAL-YDEVTDPQEIVFLAVISVLAEGACNGRLSR-------------------------------
568164518 Sulfolobus_acidocaldarius_SUSAZ           MGRTQPSFTRAVDAELAKLL----RLSE-RIGYPCFREVIVEATKRVRDFQSAL-YDEVTDPQEIVFLAVISVLAEGACNGRISR-------------------------------
330834876 Metallosphaera_cuprina_Ar-4               MGRTIPSYTASIDSFISVLE----RISK-RTG--SLEEVIEETKRRIRYFQNES-YDEDIDPQTLVILTMISVIEDE-CKRNG-------KAQGGRNQ------------------
146303843 Metallosphaera_sedula_DSM_5348            MGRTIPSYTGSVDRFISELE----RISQ-RTG--YLEDVLAETKRRIRYFQNAS-YDESIDTQTLVLVAMISVIEDM-CKGRLKDRSQ--NPSNVVNG------------------
332796087 Acidianus_hospitalis_W1                   MGRTQPSYTYSLQREIEKLE----RILS-RAS-PHLLPILERAKGKIRYFQNAS-YDEDLSPSELLFLALLSELAEG-CKKWLKDI------------------------------
15921984 Sulfolobus_tokodaii_str-_7                 MGRTQPSYTRAVDKELETVE----KIIS-RLHSPSLESLFEEVKKKVRYVQSAS-YDEFVDPYNLVYFTFIWTLAEE-CEKWKKLYS---TLIRQKEE------------------
15898292 Sulfolobus_solfataricus_P2                 MGRTQPSYTMAVNRELEKLE----RIIE-RLHSPILSLLLERVKEKVRYTQSAS-YDELVDPYNLVYFALIWALAEE-CEKWRSTYL---TLIQSREE------------------
227829768 Sulfolobus_islandicus_L-S-2-15            MGRTQPSYTMAVNRELEKLE----RIIE-RLHSPTLSLLLERVKEKVRYTQSAS-YDELIDPYNLVYFTLIWALAEE-CEKWRSTYL---TLIRSKEE------------------
227827102 Sulfolobus_islandicus_M-14-25             MGRTQPSYTMAVNRELERLE----RIIE-RLHSPTLSLLLERVKEKVRYTQSAS-YDELVDPYNLVYFTLIWALAEE-CEKWRSTYL---TLIRSKEE------------------
238619270 Sulfolobus_islandicus_M-16-4              MGRTQPSYTMAVNRELERLE----RIIE-RLHSPTLSLLLERVKEKVSYTQSAS-YDELVDPYNLVYFTLIWALAEE-CEKWRSTYL---TLIRSKEE------------------
530779616 Thermofilum_sp-_1910b                     MGRATPSVREKYLQLLNELEAEFVELLR-RERREAYIYVKKAWGE---ELGAVT-NYPNPYLLGSLLLVSVLDLEWR-LRELERRLR---DLEDEVERISSG--------------
549456042 Aeropyrum_camini_SY1_-_JCM_12091          MGRTAAPGRVLVEAEVERLR-RLIRHLRSSEDRRLLEDLLSSYRR---ILEAYR-YVPMSDPMEPVYIAMILEAARS-CRGSGGEGG---GEAHT---------------------
518652341 Ferroplasma_acidarmanus_fer1              MGRNTQSSREYVKSMEEEIL-SMRTYMK-KEDIEILKVIMSMAEK---HSNEIS-------NFNQFFAAIILELYKK-LDRYGEYNK---RHWF----------------------
297526548 Staphylothermus_hellenicus_DSM_12710      ---------MALLRELERFR-RIIARLP-RDEKARWEEILEGIED---TMSIYS-DVPITDPLEIIYFHI---LRRL-LRDDVS--------------------------------
126465180 Staphylothermus_marinus_F1                MGRTTVSYRMALLRELERFR-KIIARLP-KDEEARWEEILEDIED---TISIYS-DIPVNDPLEIIYFHI---LRRF-LREDVS--------------------------------
389860613 Thermogladius_cellulolyticus_1633         MGRTTPSLKELVNKYLERWS-SLLPLLD-PCTRAAVKEVLERADY---SASLLS-YKGVVDPLEPLVFHLLLVIAEL-KAEYEGKQC---K-------------------------
296242214 Thermosphaera_aggregans_DSM_11486         MGRSTPSLWISVSEYVERLR-KISEMLP-KDERGKILCFLEDLES---TISFCM-HTGVVDPLEVLFIHLIRKMDKE-CRGH----------------------------------
429217275 Caldisphaera_lagunensis_DSM_15908         MGRTTPSTRMVIEDEINRLL-KILEYTN--EDKDKAKEIIKEAYD---LVSYYQ-FETLSDPLEPIVLGLILYIAKR-CS------------------------------------
147919167 Methanocella_arvoryzae_MRE50              ---------MSLMKAHKRWE-GYRQTLD-KKRREIFDYIFFELAG---RREET--LVNHPDQSEAAMMNAMIEMEMR-LRELESKVQ---K-------------------------
282163291 Methanocella_paludicola_SANAE             ---------MHLMMVAKRFD-NYRLALD-RKRREVFDYIMFELAG---RREET--LVMHPNHTEAALMNAMIEMEMR-IEALEKK-------------------------------
16081571 Thermoplasma_acidophilum_DSM_1728          MGRSTPSTRQALDMIISGME-EMKKVMR-TGDAEILEELVRLGKQ---HAAEIS--YAGIDVQLGFLLAMILEVAKR-TSMPGDRTG-----------------------------
13541877 Thermoplasma_volcanium_GSS1                ----------------------MEKVMR-RSDAEVLQRLLSYGKS---HAAEVL--FSGLDAETGFIISVLIEVMKR-LSMQEDQTK-----------------------------
510882141 Salinarchaeum_sp-_Harcht-Bsk1             MGRTRATTRDRLDALEREWM-PFRRSLR-RRHQPAFDRLFEHARG---HAEAAT-QQNPADPWRGFVFAVLLAQEQA-TAAREEEIA---ALEEKLADREAEVERLAADVDALHER
433637308 Halovivax_ruber_XH-70                     MGKTNPTYRDTVRAFETEWS-TYHRALR-TQHQDQFQQLIHQVRN---FADAGG-MQNHPDPMTTHLISMLLAHECR-LTELELQLE---NPRSDPAITD----------------
433637692 Halovivax_ruber_XH-70                     MGRTNPTYRDALAQLEAEWK-PMRRALR-REYQHDFDRLFDRARG---YADAAG-YANPPDPERALVLSLLLAHEAE-IRRLNDRLD---ELERSHQSGTSETKETSMGADTGTAC
257052959 Halorhabdus_utahensis_DSM_12940           MGRTNPTFRDVLRSVEDRWA-PFRRALR-YEDQQRFDRLLGHART---HADAAG-NLNHHSPIVPVLLAISLAQERR-IDDLEARLD---ELEGEIGEQADRVDALEAQIDDFGHQ
300712716 Halalkalicoccus_jeotgali_B3               MGRTNPTYRDVLRALDNQWS-DYRRGLR-REDQQHFDQLFAYIRA---HADASG-YLNHTEPFYPALVSIDLEQEAR-LDELESRLA---QLESETDSIDSGE-------------
169237238 Halobacterium_salinarum_R1                VGRTNPTFRDALRAIEERWG-EYRRALR-RRDQPRFDQLFTYARE---HADASG-LLNHQNPMLPSLLSVDLEQESR-LDAHDERLD---DIEDAIEALRKQHDEMDDKPQPADD-
222475982 Halorubrum_lacusprofundi_ATCC_49239       MGRTNPTYRDALRAIEERWA-EFRRALR-RHDQPRFDRLFEYARE---HADASG-LLNHQNPLLLALLSIDLEQETC-LDEHEQRIA---ELETRVDQLSNRDGDTTNAGGEDNE-
55376567 Haloarcula_marismortui_ATCC_43049          MGRTNPTYRDALRAIEERWT-DFRRALR-RRDQPRFDQLFEYARE---HADASG-LLNHQNPLLPALISIDLEQEAR-LDEHEDRLA---ELENALNEADDRQQHSEAGTTEDGLH
529043603 Halorhabdus_tiamatea_SARL4B               MGRTNPTYRDALRAIEERWT-DFRRALR-RRDQPRFDQLFEYARE---HADASG-LLNHQNPLFPALISIDLEQEAR-LDEHEDRLA---ELENALNEADDRQQHSQAGTTEDRLH
222476145 Halorubrum_lacusprofundi_ATCC_49239       MGRTNPTYRDALRAIEDRWQ-DFRRALR-RRDQPRFDQLFAYVRE---HADASG-LLNHQNPLLPALLSIDLEQERR-LDEHEERLE---ELEEEIEAEENK--------------
433593175 Natrinema_pellirubrum_DSM_15624           MGRTNPTYRDALRAIEERWA-EFRRALR-RRDQPRFDRLFEYARE---HADASG-LLNHQNPLLPALFSIDLEQEAR-LDDHEERLK---EVEAAVAASDNQEAAPPDSNP-----
433593301 Natrinema_pellirubrum_DSM_15624           MGRTNPTYRDALRAIEERWA-EFRRALR-RRDQPRFDRLFEYARE---HADASG-LLNHQNPLLPALLSIDLEQEAR-LDDHEERLE---DLEAAVATSDDQEAAPPDANP-----
292653632 Haloferax_volcanii_DS2                    MGRTNPTYRDALRAIEERWA-EFRRALR-RRDQPRFDQLFEYARE---HADASG-LLNHQNPLLPALLSIDLEQEAR-LDDHEERLE---ELEAAVAARDDQESGPPDSNP-----
397771764 Natrinema_sp-_J7-2                        MGRTNPTYRDALRAIEERWV-EFRRALR-RRDQPRFDRLFEYARE---HADASG-LLNHQNPLLPALLSIDLEQEAR-LDDHEERLE---ELEAAVAARDDQESAPPDASS-----
345007177 halophilic_archaeon_DL31                  MGRTNPTYRDALRAIEERWA-EFRRALR-RRDQPRFDRLFEYARE---HADASG-LLNHQNPLLPALLSIDLEQEAR-LDDHEERLE---ELEAAVAARDDQESAPPDANP-----
433639764 Halovivax_ruber_XH-70                     MGRTNPTYRDQVRSIEERWG-DYRRALR-GQDQPHFDRLFEHGRQ---YADAAG-YQNPTDPMLALLVSIVLAQERR-ITELETQFD---ERES----------------------
488600818 Archaeoglobus_sulfaticallidus_PM70-1      MGRSVASVRLPLNDLIAKIE-RAKSLMK-KEEVIYADRIIESIKK---RYSVC--YLAFDSADKAALFAVAVELMRL-IDHADSGCLSCKERSERVAEGEEGEEKVRQA-------
488600910 Archaeoglobus_sulfaticallidus_PM70-1      MGRSVASVRIQLNGLIAKIE-RAKSLMK-REEVVYADRVIEAIKK---RYSVC--YYAFDSAEEAALFSLTIELMRL-IDHADSGCF---SVEKRNQCLAEKERCEESLHQT----
219851819 Methanosphaerula_palustris_E1-9c          ----------------------MQRALK-AEDQPAMDRLIQMLET---HAQVT--FQAFDDPLEAAFVAVLICLIKQ----GEKNAA-----------------------------
219851817 Methanosphaerula_palustris_E1-9c          MGRVFLSPRMAVQRIARNWQ-QCGVLMR-EQDQIAADYLGKMAKQ---YASEG--FYAFDDPLEAAVISALIGLVHT-------------ELDR----------------------
154149977 Methanoregula_boonei_6A8                  MGRSFLSVRQGANLISARWM-QWARKLR-PEDRKYGERLAILAKM---HSSEA--FAGCDDALEAVVFSVMVEVIRE-QERAGEEKK---DPAH----------------------
330506753 Methanosaeta_concilii_GP6                 MGRSFESVRMGVNDLSLRWS-KAGRALK-KEDQSYAKELADMAKK---HSSEA--FYALDDPLEAAVFSVLIELLKDREIYKQDPVK-----------------------------
386002264 Methanosaeta_harundinacea_6Ac             MGRTFRSVRMGSQEVADRWM-KATRTLR-KEDQIYGQRLAEMARV---HSSEG--FCALDDPLEAAAFSVLIELLKA-LDPEGDGPP---GGLKGGKSVDKRVDDGGVKKPDVDL-
408405375 Candidatus_Nitrososphaera_gargensis_Ga9-2 ---------MALMEEESEWK-GFRKCLE-RKERKILDGMFDKSRL---YIPSCM-YSANPIVIYPIFLSMLLHHYRE-LSEIVNRVE---QLTGERYDTIPK--------------
557694811 Candidatus_Caldiarchaeum_subterraneum     ---------------MRGWS-GFREALR-REEREAFDNLVNQAFR---YVHAGT-MNPLRRAFDNLTMSLLLSHEER-LRQLEETLR---KTMQIDNRYPTLNHFLEK--------
408405376 Candidatus_Nitrososphaera_gargensis_Ga9-2 --MTLFPNDDIITQIIGSWQ-SYADVLN-AEDRIAFDNMMKKCYK---YSRSIE-AKAKPFENEALFMAMLLEQEKI-ILWLLAKIE---KLEKGKVEKP----------------
```

Bacteria

```
383784776 Leptospirillum_ferrooxidans_C2-3          MGKSTPTFRELVQLEKQLWN-NYTHSLS-REEKKRFERLFNKALF---LSPAAGEMAGKKDPLMLMIMNIFMEMESR-IEKLEEEHS---GKIFPEER------------------
410478545 Leptospirillum_ferriphilum_ML-04          MGRTLETFTQKIDRIRSEWS-LFRRALR-REDQILLDTLFDHARL---HAQAGS-YASPPDPFSAILLSILIEERKA-RLAQEERIR---ALEQRLR-------------------
225851069 Persephonella_marina_EX-H1                MGKTIPSGTQIIKLSKNELS-LFRKALR-KEDKKIFDRLFSYAKF---HSQAVG-SSGSINPVESIIFSILIEMEKE-IQELK---N---GKKKASDP------------------
188997123 Sulfurihydrogenibium_sp-_YO3AOP1          MGKTIPSATGIVSLKQKEYS-EFRKALR-KEDKEVLDKLFAYAKF---HSPAIG-CSNQFFPMESIILSMLIEMQKE-IDRLKAQIE---KNENISNP------------------
183221541 Leptospira_biflexa_serovar_Patoc_Paris    MGRTISPYSRQMLQIEENLS-DFRRALR-KADQEIFDDLIRIAKL---QVQAGV-MASLPYPIDSMLLSMMIELKKE-LNELSELKK---KLSKELNLS-----------------
392401637 Turneriella_parva_DSM_21527               MGRTVVPYSLVIEQVMHRFD-NYRRTLR-REDREAFDELMRVAKM---QVQAGV-MAQHPNAFDSMSMAMLVQLKRE-INELKKNIKSQGELYAGVETQ-----------------
328953848 Desulfobacca_acetoxidans_DSM_11109        MGRTVIPYTQIVDQERSRWQ-KFRRALR-REDQAHFDRLFELARR---HTQAGV-YASQPWPMEVILMSMLLEHQKA-LAVLAERLR---RLEEGGNGDPGLAL--------------
```

Supplementary Figure S2:

HHpred alignments and statistics for two most divered inactivated RecA/RadA families associated with PolB2

A. Results for Mhar_1579 (Methanosaeta harundinacea 6Ac), arCOG07763 as a query

```
>2w0m_A SSO2452; RECA, SSPF, unknown FUN; 2.0A {Sulfolobus solfataricus P2}
  Probab=92.54  E-value=4  Score=32.67  Aligned_cols=152  Identities=16%  Similarity=0.145  Sum_probs=0.0

  Q ss_pred              ecCCceEEEEECcH----HHHHHHHhhccccccceeEEEEeCCccc----------------------hhhcCCCCccceE
  Q Fri_Apr_11_22:    11 LAPHAFNVIISPE----EVLIASLNGQASLQRVQVLYVSGNSSR----------------------ILDKLNRRFTELD   63 (171)
  Q Consensus         11 l~~~tF~~~vaP~~~~~~~~~~aL~~~~~~~~r~~~LYi~Gn~sr----------------------iL~~~~r~~~~~e   63 (171)
                         |++|+++++.+|+    ..+..+....-.....++|++...+.                       ..+..........
  T Consensus         20 l~~G~~~l~~G~~G~GKT~~a~~~~~~~~~~~g~~v~~~s~e~~~~~~~~~~~~~~g~~~~~~~~~~~~~~~d~~~~~~~~~~   99 (235)
  T 2w0m_A            20 IPQGFFIAITGEPGTGKTIFSLHFIAKGLRDGDPCIYVTTEESRDSIIRQAKQFNWDFEEYIEKKLIIIDALMKEKEDQW   99 (235)
  T ss_dssp              EETTCEEEEECSTTSSHHHHHHHHHHHHHHHTCCEEEEESSSCHHHHHHHHHHTTCCCGGGBTTTEEEEECCC----CTT
  T ss_pred              CcCCeEEEEEEcCCCCHHHHHHHHHHHHHHHhccccceeeeccCCHHHHHHHHHHcCCChHHHHhcCccchhhhhhhhhhcc

  Q ss_pred              EEEeccHHHHHHHHHh------CCccEEEEEeCHHHhcCcchhhHHHHHHHHHHHhcccCceEEEEecCCChHHHH----H
  Q Fri_Apr_11_22:    64 VRRAFTAHQLLTILKE------AYQTLIIFEHDPSLFEDGADMAEYVGQALKEASASGGSAVLLYSPSTDRHLDE----I  133 (171)
  Q Consensus         64 vrrafta~qLL~iL~e------~~~~i~vEhDp~lFe~~~d~~~~~g~alr~~~~~r~a~VlL~s~~~D~~~~~----i  133 (171)
                         .....+..+++.-+.+        ...+|+|=+=-.++.+.+.....+-..|+..+++.+.+|++.+...+..-+.    +
  T Consensus        100 ~~~~~~~~~~~~i~~~~~~~~~~~~~vviDsl~~l~~~~~~~~~~~~~~~~l~~~~~k~~~~~vllv~~~~~~~~~~~~~~~~l   179 (235)
  T 2w0m_A           100 SLVNLTPEELVNKVIEAKQKLGYGKARLVIDSVSALFLDKPAMARKISYYLKRVLNKWNFTIYATSQYAITTSQAFGFGV   179 (235)
  T ss_dssp              BCSSCCHHHHHHHHHHHHHHHCSSCEEEEEEETGGGSSSCGGGHHHHHHHHHHHHHHTTEEEEEEEC-----------CH
  T ss_pred              ccccCCHHHHHHHHHHHHHHHhccCCceEEEEcchHHhhcCHHHHHHHHHHHHHHHHHcCCeEEEEEcccCCccccCCcce

  Q ss_pred              HhhCCeEEEEeeccccccc--------cCcCccccCCCc
  Q Fri_Apr_11_22:   134 SKFADRIFVFNEVQKCVY--------KGASKKVKPGP  162 (171)
  Q Consensus        134 ~~~AdRvi~v~e~~~~~~~~--------kg~~~~~~~~~~  162 (171)
                         +..||-||.+........          |.|+..+.++.
  T Consensus        180 ~~~aD~vi~l~~~~~~~~~~~~R~l~v~K~R~~~~~~~~~  216 (235)
  T 2w0m_A           180 EHVADGIIRFRRMIRNGELHRYILIEKMRQTDHDKHV  216 (235)
  T ss_dssp              HHHCSEEEEEEEEEETTEEEEEEEEEEETTCCCCCSC
  T ss_pred              eeeccEEEEEEEEccCCeEEEEEEEEEECCCCCCCCeE
```

Walker A motif

Walker B motif

B. Results for protein LFML04_0992 (Leptospirillum ferriphilum ML-04), as a query

```
>PF08423 Rad51:  Rad51;  InterPro: This domain is found at the C terminus of the DNA repair and
  recombination protein Rad51. It is critical for DNA binding []. Rad51 is a homologue of the bacterial RecA protein.
  Rad51 and RecA share a core ATPase domain.; PDB: 2ZUC_A 2Z43_C 2ZUD_B 2DFL_A 2ZUB_A 2BKE_A 2KZ3_A 2CVH_B 2CVF
_B 1SZP_D ....
  Probab=99.92  E-value=5.7e-24  Score=181.03  Aligned_cols=138  Identities=27%  Similarity=0.374  Sum_probs=0.0

  Q ss_pred              CCcceEEeecccc-chHHHHHHHHHHHHhC------CCcEEEEeCCCCCcCHHHHHHHHHHcCCCHHHHHhhhheeeecCHH
  Q Fri_Apr_25_21:    13 LPSPLIVFVPRSS-SPDALSFFLAKELLR------GQKAILLSADNRFRPDLLMQKAREAGQAPESVLSRLLISRAFTIH   85 (178)
  Q Consensus         13 ~~~~l~v~~~r~~~~~~~~~~~~la~el~~~~~~~~G~kv~~IDte~tFrPeRI~~ia~r~gldpe~vL~nI~vaRAfT~h   85 (178)
                         ..+..+|.++-.+ -+-+...+.+...+.       |++|+|||||++|+|+|+||.||+++++++++++++|+||+|.|+||.+
  T Consensus         37 ~g~itli~G~~gsGKTql~l~l~~~~~~l~~~~~~g~~~~~vvyidTe~~f~~~Rl~~i~~~~~~~~~~~~~~~l~~I~v~~~~~~~   116 (256)
  T PF08423_consen    37 TGSITEIVGESGSGKTQLCLQLAVNVQLPEEIGGLGGKVVYIDTEGTFSPERLQQIAERFGLDPEEILDNIFVIRVFDLE   116 (256)
  T ss_dssp              TTSEEEEEESTTSSHHHHHHHHHHHTTSGGCTTSSSSEEEEEESSSSS-HHHHHHHHHHTTS-HHHHHHTEEEEE-SSHH
  T ss_pred              CCcEEEEEEecccccchHHHHHHHHhhcccccccCCCCceEEEeCCCCCCCHHHHHHHhhcccccccchhhhceeeeecCCHH

  Q ss_pred              HHHHHHHHHHHHHHHHhhcCCCcEEEECCHHHhhcccccc---chHHHHHHHHHHHHHHHHHHHHHhhcCeeEEEE
  Q Fri_Apr_25_21:    86 QLQETISDRLEPAIQFSGATLVVVTGILPLFADEAV---ETREAFRVLEILLESLKRLARSDRRILAPV  151 (178)
  Q Consensus         86 Q~~~LI~erL~~ei~e~~~~LIVVDsi~alF~DeqV------E~~~LL~~~L~~L~~lArs~~~~~~~~~~~~~  151 (178)
                         |+.++| +.+...+.+.+++|||||||+|++|+.+..    ...|++.+|.+++..|+++|+..+++|+++
  T Consensus        117 ~l~~~L~~~l~~~l~~~~~~ikLIVIDSIaalfr~e~~~~~~~~~~~~R~~~L~~~~~~~~L~~lA~~~~~iaVvvT  184 (256)
  T PF08423_consen   117 ELLELL-EQLPKLLSESKIKLIVIDSIAALFRSEFSGRGDLAERQRMLARLARILKRLARKYNIAVVVT  184 (256)
  T ss_dssp              HHHHHH-HHHHHHHHHSCEEEEEEETSHHHHHHHSGSTTTHHHHHHHHHHHHHHHHHHHHHHHHTT-EEEEE
  T ss_pred              HHHHHH-HHHHHhhcccceEEEEeecchHHHHHHHHcccchhhHHHHHHHHHHHHHHHHHHHHHhCCceEEee
```

Walker A motif

Walker B motif

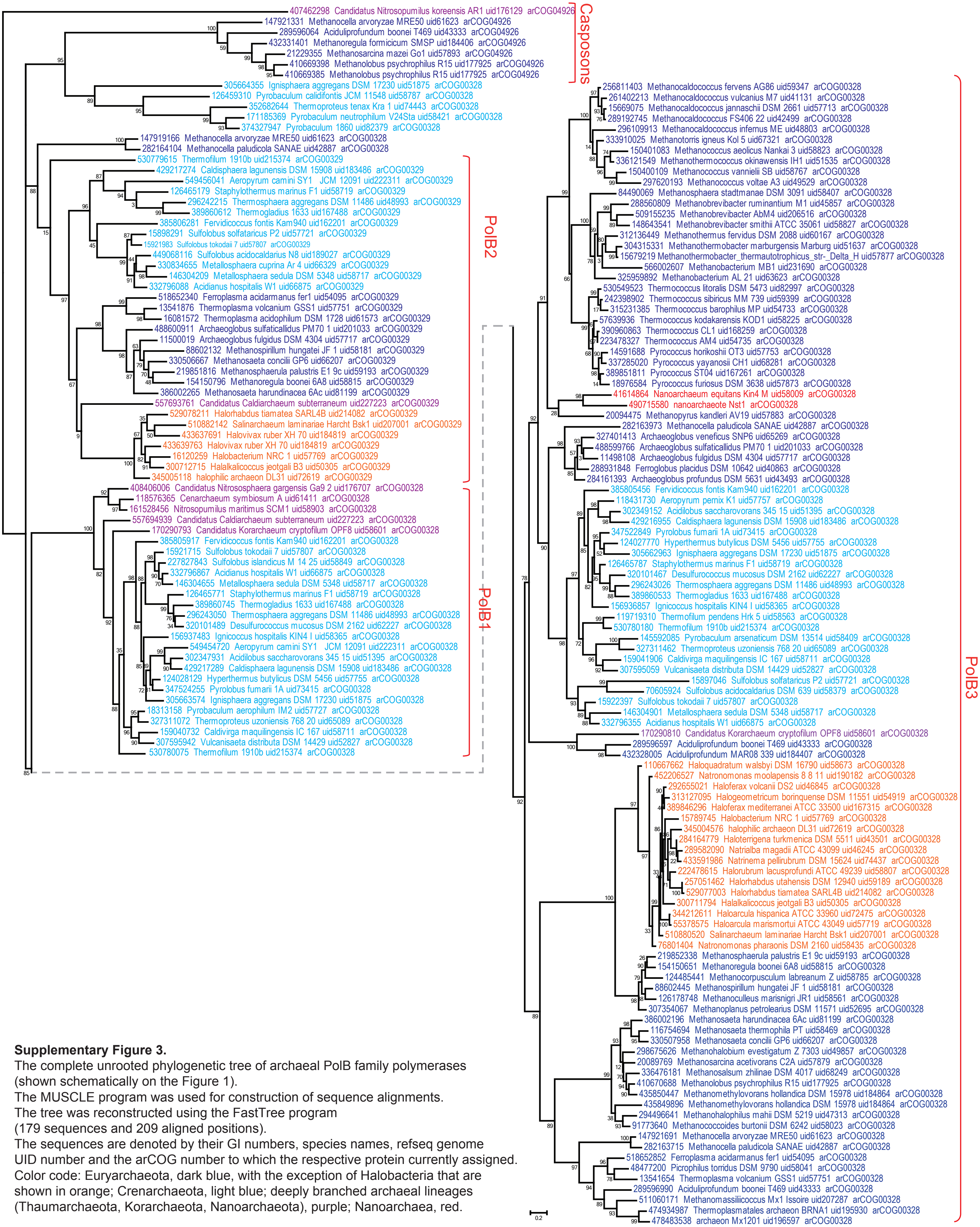

Supplementary Figure 3.
The complete unrooted phylogenetic tree of archaeal PolB family polymerases (shown schematically on the Figure 1).
The MUSCLE program was used for construction of sequence alignments.
The tree was reconstructed using the FastTree program (179 sequences and 209 aligned positions).
The sequences are denoted by their GI numbers, species names, refseq genome UID number and the arCOG number to which the respective protein currently assigned.
Color code: Euryarchaeota, dark blue, with the exception of Halobacteria that are shown in orange; Crenarchaeota, light blue; deeply branched archaeal lineages (Thaumarchaeota, Korarchaeota, Nanoarchaeota), purple; Nanoarchaea, red.

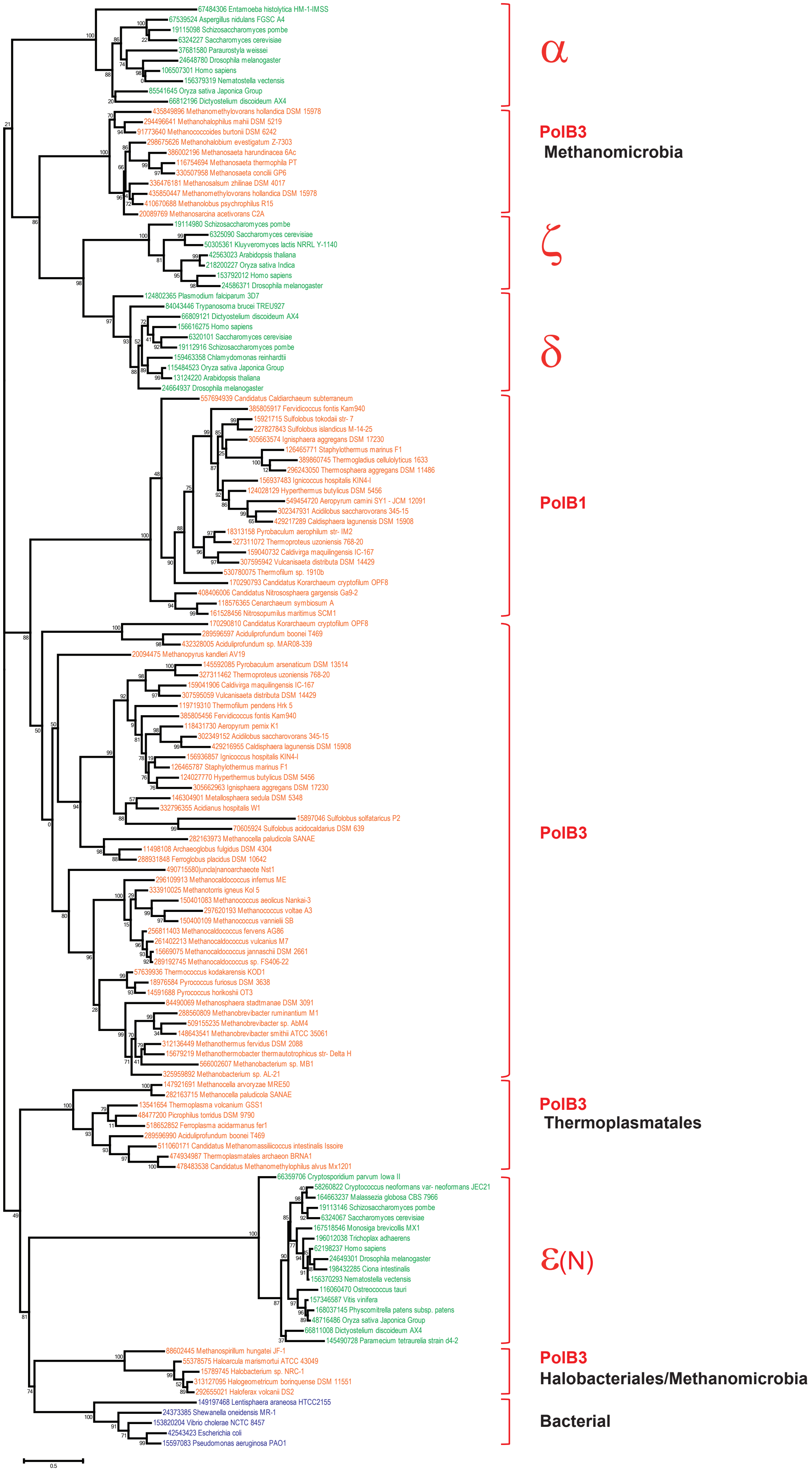

Supplementary Figure S4.

The complete unrooted phylogenetic tree of active polymerases of B family (shown schematically on the Fiogure 4B.

The MUSCLE program was used for construction of multiple sequence alignments.

The tree was reconstructed using the FastTree program (84 sequences and 380 aligned positions).

The sequences are denoted by their GI numbers and species names.

Supplementary Table S1. Presense and absense of arCOGs related to DNA replication and repair polymerases in archaea
Number of proteins in arCOG by genomes (details and explanations are in ftp://ftp.ncbi.nih.gov/pub/wolf/COGs/arCOG/)

		DNA replication polymerases				PolB2 components					DNA repair polymerases	
arCOG		arCOG04926	arCOG00331	arCOG04455	arCOG04447	arCOG00329	arCOG07300	arCOG07763	arCOG04294	arCOG08101	arCOG04582	arCOG00305
Gene		PolB	PolB	DP2	DP1	PolB			RecA		DinP	POL4
Annotation		DNA polymerase elongation subunit (family B)	DNA polymerase elongation subunit (family B), C-terminal domain	Archaeal DNA polymerase II, small subunit/DNA polymerase delta, subunit B	Archaeal DNA polymerase II, large subunit	DNA polymerase elongation subunit (family B), inactivated	Uncharacterized conserved protein associated with inactivated PolB-like polymerase	Inactivated RecA-like ATPase associated with anactivated PolB gene	RecA/RadA recombinase, inactivated	Inactivated RecA-like ATPase associated with anactivated PolB gene	Nucleotidyltransferase/DNA polymerase involved in DNA repair	DNA polymerase IV (family X)
Number of organisms		9	16	117	117	55	50	7	29	10	75	55
Number of proteins (of fragments)		18	16	126	163	64	60	14	29	16	86	67
Organisms	Acidilobus_saccharovorans_345_15_uid513	0	0	0	0	0	0	0	0	0	0	0
	Caldisphaera_lagunensis_DSM_15908_uid1	0	0	0	0	1	1	0	1	0	0	0
	Aeropyrum_camini_SY1___JCM_12091_uid	0	0	0	0	1	1	0	1	0	0	0
	Aeropyrum_pernix_K1_uid57757	0	0	0	0	1	0	0	1	0	0	0
	Desulfurococcus_fermentans_DSM_16532_	0	0	0	0	0	0	0	0	0	0	0
	Desulfurococcus_kamchatkensis_1221n_uid	0	0	0	0	0	0	0	0	0	0	0
	Desulfurococcus_mucosus_DSM_2162_uid6	0	0	0	0	0	0	0	0	0	0	0
	Ignicoccus_hospitalis_KIN4_I_uid58365	0	0	0	0	0	0	0	0	0	0	0
	Ignisphaera_aggregans_DSM_17230_uid51	0	0	0	0	0	0	0	0	0	0	0
	Staphylothermus_hellenicus_DSM_12710_u	0	0	0	0	1	1	0	1	0	0	0
	Staphylothermus_marinus_F1_uid58719	0	0	0	0	1	1	0	1	0	0	0
	Thermogladius_1633_uid167488	0	0	0	0	1	1	0	1	0	0	0
	Thermosphaera_aggregans_DSM_11486_ui	0	0	0	0	1	1	0	1	0	0	0
	Hyperthermus_butylicus_DSM_5456_uid57	0	0	0	0	0	0	0	0	0	0	0
	Pyrolobus_fumarii_1A_uid73415	0	0	0	0	0	0	0	0	0	0	0
	Fervidicoccus_fontis_Kam940_uid162201	0	0	0	0	1	0	0	1	0	0	0
	Acidianus_hospitalis_W1_uid66875	0	0	0	0	1	1	0	1	0	1	0
	Metallosphaera_cuprina_Ar_4_uid66329	0	0	0	0	1	1	0	1	0	1	0
	Metallosphaera_sedula_DSM_5348_uid587	0	0	0	0	1	1	0	1	0	1	0
	Sulfolobus_acidocaldarius_DSM_639_uid58	0	0	0	0	0	0	0	1	0	1	0
	Sulfolobus_acidocaldarius_N8_uid189027	0	0	0	0	1	1	0	1	0	1	0
	Sulfolobus_acidocaldarius_Ron12_I_uid189	0	0	0	0	1	1	0	1	0	1	0
	Sulfolobus_acidocaldarius_SUSAZ_uid23225	0	0	0	0	1	1	0	1	0	1	0
	Sulfolobus_islandicus_HVE10_4_uid162067	0	0	0	0	1	1	0	1	0	1	0
	Sulfolobus_islandicus_L_D_8_5_uid43679	0	0	0	0	1	1	0	1	0	1	0
	Sulfolobus_islandicus_L_S_2_15_uid58871	0	0	0	0	1	1	0	1	0	1	0
	Sulfolobus_islandicus_LAL14_1_uid197216	0	0	0	0	1	1	0	1	0	1	0
	Sulfolobus_islandicus_M_14_25_uid58849	0	0	0	0	1	1	0	1	0	1	0
	Sulfolobus_islandicus_M_16_27_uid58851	0	0	0	0	1	1	0	1	0	1	0
	Sulfolobus_islandicus_M_16_4_uid58841	0	0	0	0	1	1	0	1	0	1	0
	Sulfolobus_islandicus_REY15A_uid162071	0	0	0	0	1	1	0	1	0	1	0
	Sulfolobus_islandicus_Y_G_57_14_uid5892	0	0	0	0	1	1	0	1	0	1	0
	Sulfolobus_islandicus_Y_N_15_51_uid5882	0	0	0	0	1	1	0	1	0	1	0
	Sulfolobus_solfataricus_98_2_uid167998	0	0	0	0	1	1	0	1	0	1	0
	Sulfolobus_solfataricus_P2_uid57721	0	0	0	0	1	1	0	1	0	1	0
	Sulfolobus_tokodaii_7_uid57807	0	0	0	0	1	1	0	1	0	1	0
	Thermofilum_pendens_Hrk_5_uid58563	0	0	0	0	0	0	0	0	0	0	0
	Thermofilum_1910b_uid215374	0	0	0	0	1	1	0	1	0	0	0
	Caldivirga_maquilingensis_IC_167_uid5871	0	0	0	0	0	0	0	0	0	0	0
	Pyrobaculum_aerophilum_IM2_uid57727	0	0	0	0	0	0	0	0	0	0	0
	Pyrobaculum_arsenaticum_DSM_13514_uid	0	0	0	0	0	0	0	0	0	0	0
	Pyrobaculum_calidifontis_JCM_11548_uid5	0	0	0	0	0	0	0	0	0	0	0
	Pyrobaculum_islandicum_DSM_4184_uid58	0	0	0	0	0	0	0	0	0	0	0
	Pyrobaculum_neutrophilum_V24Sta_uid58	0	0	0	0	0	0	0	0	0	0	0
	Pyrobaculum_oguniense_TE7_uid84411	0	0	0	0	0	0	0	0	0	0	0
	Pyrobaculum_1860_uid82379	0	0	0	0	0	0	0	0	0	0	0
	Thermoproteus_tenax_Kra_1_uid74443	0	0	0	0	0	0	0	0	0	0	0
	Thermoproteus_uzoniensis_768_20_uid650	0	0	0	0	0	0	0	0	0	0	0
	Vulcanisaeta_distributa_DSM_14429_uid52	0	0	0	0	0	0	0	0	0	0	0
	Vulcanisaeta_moutnovskia_768_28_uid636	0	0	0	0	0	0	0	0	0	0	0
	Archaeoglobus_fulgidus_DSM_4304_uid577	0	0	1	1	1	FS	1	0	0	0	0
	Archaeoglobus_profundus_DSM_5631_uid4	0	0	1	1	0	0	0	0	0	0	0
	Archaeoglobus_sulfaticallidus_PM70_1_uid	0	0	1	1	1	2	2	0	0	0	0
	Archaeoglobus_veneficus_SNP6_uid65269	0	0	1	2	0	0	0	0	0	0	0
	Ferroglobus_placidus_DSM_10642_uid4086	0	0	1	1	0	0	0	0	0	0	0
	Halalkalicoccus_jeotgali_B3_uid50305	0	0	1	1	1	1	0	0	1	1	1
	Haloarcula_hispanica_ATCC_33960_uid7247	0	0	1	2	0	0	0	0	0	1	2
	Haloarcula_hispanica_N601_uid230920	0	0	1	2	0	0	0	0	0	1	2
	Haloarcula_marismortui_ATCC_43049_uid5	0	0	1	2	1	1	0	0	1	1	1
	Halobacterium_salinarum_R1_uid61571	0	0	1	2	3	2	0	0	2	1	0
	Halobacterium_NRC_1_uid57769	0	0	1	2	3	FS	0	0	0	1	0
	Haloferax_mediterranei_ATCC_33500_uid1	0	0	1	2	0	0	0	0	0	1	1
	Haloferax_volcanii_DS2_uid46845	0	0	1	1	1	1	0	0	1	1	1
	Halogeometricum_borinquense_DSM_1155	0	0	1	1	0	0	0	0	0	1	1
	Halomicrobium_mukohataei_DSM_12286_u	0	0	1	2	0	0	0	0	0	1	2
	Halopiger_xanaduensis_SH_6_uid68105	0	0	1	1	0	0	0	0	0	1	2
	Haloquadratum_walsbyi_C23_uid162019	0	0	1	2	0	0	0	0	0	1	1
	Haloquadratum_walsbyi_DSM_16790_uid5	0	0	1	3	0	0	0	0	0	1	1
	Halorhabdus_tiamatea_SARL4B_uid214082	0	0	1	1	2	1	0	0	1	1	2
	Halorhabdus_utahensis_DSM_12940_uid59	0	0	1	1	1	1	0	0	0	1	2
	Halorubrum_lacusprofundi_ATCC_49239_ui	0	0	1	2	1	2	0	0	2	1	2
	Haloterrigena_turkmenica_DSM_5511_uid4	0	0	1	1	0	0	0	0	0	1	2
	Halovivax_ruber_XH_70_uid184819	0	0	1	1	2	3	0	0	2	1	1
	Natrialba_magadii_ATCC_43099_uid46245	0	0	1	3	0	0	0	0	0	1	2
	Natrinema_pellirubrum_DSM_15624_uid74	0	0	1	3	1	2	0	0	2	1	1
	Natrinema_J7_uid171337	0	0	1	1	3	1	0	0	0	1	1
	Natronobacterium_gregoryi_SP2_uid74439	0	0	1	1	0	0	0	0	0	1	1
	Natronococcus_occultus_SP4_uid184863	0	0	1	2	0	0	0	0	0	1	1
	Natronomonas_moolapensis_8_8_11_uid19	0	0	1	1	0	0	0	0	0	2	1
	Natronomonas_pharaonis_DSM_2160_uid5	0	0	1	1	0	0	0	0	0	1	1
	Salinarchaeum_laminariae_Harcht_Bsk1_ui	0	0	1	2	1	1	0	0	1	1	1
	halophilic_archaeon_DL31_uid72619	0	0	1	3	2	3	0	0	3	1	1
	Methanobacterium_AL_21_uid63623	0	1	1	1	0	0	0	0	0	0	1
	Methanobacterium_MB1_uid231690	0	1	1	1	0	0	0	0	0	0	1
	Methanobacterium_SWAN_1_uid67359	0	0	1	1	0	0	0	0	0	0	1
	Methanobrevibacter_ruminantium_M1_uid	0	1	1	1	0	0	0	0	0	0	0
	Methanobrevibacter_smithii_ATCC_35061_	0	1	1	1	0	0	0	0	0	0	0
	Methanobrevibacter_AbM4_uid206516	0	1	1	1	0	0	0	0	0	0	0
	Methanosphaera_stadtmanae_DSM_3091_	0	1	1	1	0	0	0	0	0	0	0
	Methanothermobacter_marburgensis_Mar	0	1	1	1	0	0	0	0	0	0	1
	Methanothermobacter_thermautotrophicu	0	1	1	1	0	0	0	0	0	0	1
	Methanothermus_fervidus_DSM_2088_uid	0	1	1	2	0	0	0	0	0	0	0
	Methanocaldococcus_fervens_AG86_uid59	0	0	1	1	0	0	0	0	0	0	0
	Methanocaldococcus_infernus_ME_uid488	0	0	1	2	0	0	0	0	0	0	0
	Methanocaldococcus_jannaschii_DSM_2661	0	0	2	1	1	0	0	0	0	0	0
	Methanocaldococcus_FS406_22_uid42499	0	0	1	2	0	0	0	0	0	0	0
	Methanocaldococcus_vulcanius_M7_uid411	0	0	1	2	0	0	0	0	0	0	0
	Methanotorris_igneus_Kol_5_uid67321	0	0	1	2	0	0	0	0	0	0	0
	Methanococcus_aeolicus_Nankai_3_uid588	0	0	1	2	0	0	0	0	0	0	0
	Methanococcus_maripaludis_C5_uid58741	0	0	1	1	0	0	0	0	0	0	0
	Methanococcus_maripaludis_C6_uid58947	0	0	1	1	0	0	0	0	0	0	0
	Methanococcus_maripaludis_C7_uid58847	0	0	1	1	0	0	0	0	0	0	0
	Methanococcus_maripaludis_S2_uid58035	0	0	1	1	0	0	0	0	0	0	0
	Methanococcus_maripaludis_X1_uid70729	0	0	1	1	0	0	0	0	0	0	0
	Methanococcus_vannielii_SB_uid58767	0	0	1	1	0	0	0	0	0	0	0
	Methanococcus_voltae_A3_uid49529	0	0	1	1	0	0	0	0	0	0	1
	Methanothermococcus_okinawensis_IH1_u	0	0	1	2	0	0	0	0	0	0	0
	Methanocella_arvoryzae_MRE50_uid61623	1	0	1	1	0	1	0	0	0	0	1
	Methanocella_conradii_HZ254_uid157911	0	0	1	1	0	0	0	0	0	0	1
	Methanocella_paludicola_SANAE_uid42887	0	0	1	1	0	1	0	0	0	0	1
	Methanocorpusculum_labreanum_Z_uid58	0	0	1	1	0	0	0	0	0	0	0
	Methanoculleus_bourgensis_MS2_uid1713	0	0	1	2	0	0	0	0	0	1	2
	Methanoculleus_marisnigri_JR1_uid58561	0	0	1	2	0	0	0	0	0	1	2
	Methanoplanus_petrolearius_DSM_11571_	0	0	1	2	0	0	0	0	0	1	0

Methanoregula_boonei_6A8_uid58815	0	0	1	2	1	1	2	0	0	1	0
Methanoregula_formicicum_SMSP_uid1844	1	0	1	2	1	0	1	0	0	1	0
Methanosphaerula_palustris_E1_9c_uid591	0	0	1	2	1	2	0	0	0	0	2
Methanospirillum_hungatei_JF_1_uid58181	0	0	1	2	1	0	4	0	0	1	0
Methanosaeta_concilii_GP6_uid66207	0	0	1	1	1	1	3	0	0	1	1
Methanosaeta_harundinacea_6Ac_uid8119	0	0	1	1	1	1	1	0	0	0	1
Methanosaeta_thermophila_PT_uid58469	0	0	1	1	0	0	0	0	0	0	1
Methanococcoides_burtonii_DSM_6242_uic	7	0	0	1	0	0	0	0	0	2	0
Methanohalobium_evestigatum_Z_7303_ui	0	0	1	1	0	0	0	0	0	1	1
Methanohalophilus_mahii_DSM_5219_uid4	0	0	1	1	0	0	0	0	0	2	1
Methanolobus_psychrophilus_R15_uid1779	3	0	1	1	0	0	0	0	0	2	1
Methanomethylovorans_hollandica_DSM_1	0	0	1	1	0	0	0	0	0	2	1
Methanosalsum_zhilinae_DSM_4017_uid68	0	0	1	1	0	0	0	0	0	2	1
Methanosarcina_acetivorans_C2A_uid5787	0	0	2	1	0	0	0	0	0	2	1
Methanosarcina_barkeri_Fusaro_uid57715	0	0	2	1	0	0	0	0	0	2	1
Methanosarcina_mazei_Go1_uid57893	1	0	2	1	0	0	0	0	0	2	1
Methanosarcina_mazei_Tuc01_uid190185	1	0	2	1	0	0	0	0	0	2	1
Methanopyrus_kandleri_AV19_uid57883	0	0	2	2	0	0	0	0	0	0	0
Pyrococcus_abyssi_GE5_uid62903	0	0	1	2	0	0	0	0	0	0	0
Pyrococcus_furiosus_COM1_uid169620	0	0	1	1	0	0	0	0	0	0	0
Pyrococcus_furiosus_DSM_3638_uid57873	0	0	2	1	0	0	0	0	0	0	0
Pyrococcus_horikoshii_OT3_uid57753	0	0	2	2	0	0	0	0	0	0	0
Pyrococcus_NA2_uid66551	0	0	1	2	0	0	0	0	0	0	0
Pyrococcus_ST04_uid167261	0	1	1	2	0	0	0	0	0	0	0
Pyrococcus_yayanosii_CH1_uid68281	0	1	1	1	0	0	0	0	0	0	0
Thermococcus_barophilus_MP_uid54733	0	0	1	2	0	0	0	0	0	0	0
Thermococcus_gammatolerans_EJ3_uid593	0	0	1	2	0	0	0	0	0	0	1
Thermococcus_kodakarensis_KOD1_uid582	0	0	2	2	0	0	0	0	0	0	0
Thermococcus_litoralis_DSM_5473_uid829	0	1	1	1	0	0	0	0	0	0	0
Thermococcus_onnurineus_NA1_uid59043	0	0	1	2	0	0	0	0	0	0	0
Thermococcus_sibiricus_MM_739_uid5939	0	1	1	2	0	0	0	0	0	0	0
Thermococcus_4557_uid70841	0	0	1	2	0	0	0	0	0	0	0
Thermococcus_AM4_uid54735	0	1	1	1	0	0	0	0	0	0	0
Thermococcus_CL1_uid168259	0	1	1	1	0	0	0	0	0	0	0
Methanomassiliicoccus_Mx1_Issoire_uid20	0	0	1	1	0	0	0	0	0	0	1
Ferroplasma_acidarmanus_fer1_uid54095	0	0	1	1	1	1	0	0	0	1	1
Picrophilus_torridus_DSM_9790_uid58041	0	0	1	1	0	0	0	0	0	1	0
Thermoplasma_acidophilum_DSM_1728_ui	0	0	1	1	1	1	0	0	0	0	1
Thermoplasma_volcanium_GSS1_uid57751	0	0	1	1	1	1	0	0	0	0	1
Thermoplasmatales_archaeon_BRNA1_uid1	0	0	1	1	0	0	0	0	0	1	0
Aciduliprofundum_boonei_T469_uid43333	1	0	1	1	0	0	0	0	0	0	0
Aciduliprofundum_MAR08_339_uid184407	0	0	1	1	0	0	0	0	0	0	0
archaeon_Mx1201_uid196597	0	0	1	2	0	0	0	0	0	0	0
Candidatus_Korarchaeum_cryptofilum_OPF	0	0	1	1	0	0	0	0	0	0	0
Nanoarchaeum_equitans_Kin4_M_uid5800	0	1	1	1	0	0	0	0	0	0	0
nanoarchaeote_Nst1	0	0	1	1	0	0	0	0	0	0	0
Cenarchaeum_symbiosum_A_uid61411	0	0	1	1	0	0	0	0	0	1	0
Nitrosoarchaeum_limnia_SFB1	0	0	1	1	0	0	0	0	0	1	0
Candidatus_Nitrosopumilus_koreensis_AR1_	1	0	1	1	0	0	0	0	0	1	0
Nitrosoarchaeum_koreensis_MY1_MY1	0	0	1	1	0	0	0	0	0	1	0
Candidatus_Nitrosopumilus_AR2_uid17613	0	0	1	0	0	0	0	0	0	1	0
Nitrosopumilus_maritimus_SCM1_uid58903	0	0	1	1	0	0	0	0	0	1	0
Candidatus_Nitrososphaera_gargensis_Ga9_	2	0	1	1	1	2	0	0	0	2	1
Candidatus_Caldiarchaeum_subterraneum_	0	0	1	1	1	1	0	0	0	1	0

Supplementary Table S2. Selected top PSIBLAST hits for PolB3 from Methanosarcina mazei Go1

Refseq ID for the BLAST hit	Refseq annotation	Organism (archaea - blue, eukaryotes - green)	Bit Score
NP_634028.1	DNA polymerase delta catalytic subunit	Methanosarcina mazei Go1	1849
YP_007490687.1	Archaeal DNA polymerase I	Methanosarcina mazei Tuc01	1834
NP_615844.1	DNA-directed DNA polymerase	Methanosarcina acetivorans C2A	1653
YP_305298.1	replicative DNA polymerase I	Methanosarcina barkeri str. Fusaro	1551
YP_006923059.1	DNA polymerase Pol2	Methanolobus psychrophilus R15	1157
WP_023845458.1	DNA polymerase elongation subunit (family B)	Methanolobus tindarius	1144
YP_007312033.1	DNA polymerase elongation subunit (family B)	Methanomethylovorans hollandica DSM 15978	1117
YP_566332.1	replicative DNA polymerase I	Methanococcoides burtonii DSM 6242	1114
YP_003727376.1	DNA polymerase Pol2	Methanohalobium evestigatum Z-7303	1101
YP_004615322.1	DNA polymerase Pol2	Methanosalsum zhilinae DSM 4017	1070
YP_003543134.1	replicative DNA polymerase I	Methanohalophilus mahii DSM 5219	1061
YP_007301837.1	DNA polymerase elongation subunit (family B)	Methanomethylovorans hollandica DSM 15978	881
YP_005920495.1	DNA polymerase	Methanosaeta harundinacea 6Ac	857
YP_004384386.1	DNA polymerase 2	Methanosaeta concilii GP6	848
YP_843812.1	DNA polymerase Pol2	Methanosaeta thermophila PT	805
XP_005468385.1	PREDICTED: LOW QUALITY PROTEIN: DNA polymerase delta c	Oreochromis niloticus	405
XP_006809208.1	PREDICTED: DNA polymerase delta catalytic subunit-like	Neolamprologus brichardi	404
XP_004573763.1	PREDICTED: DNA polymerase delta catalytic subunit-like	Maylandia zebra	402
XP_007020936.1	DNA binding,nucleotide bindin isoform 3	Theobroma cacao	400
XP_005753466.1	PREDICTED: LOW QUALITY PROTEIN: DNA polymerase delta c	Pundamilia nyererei	401
XP_005945293.1	PREDICTED: LOW QUALITY PROTEIN: DNA polymerase delta c	Haplochromis burtoni	401
XP_004502958.1	PREDICTED: DNA polymerase delta catalytic subunit-like	Cicer arietinum	400
NP_001034899.1	DNA polymerase delta catalytic subunit	Danio rerio	400
XP_007020934.1	DNA binding,nucleotide bindin isoform 1	Theobroma cacao	400
XP_007256346.1	PREDICTED: DNA polymerase delta catalytic subunit-like isofo	Astyanax mexicanus	400
XP_007562915.1	PREDICTED: DNA polymerase delta catalytic subunit	Poecilia formosa	399
XP_004143345.1	PREDICTED: DNA polymerase delta catalytic subunit-like	Cucumis sativus	397
XP_003577757.1	PREDICTED: DNA polymerase delta catalytic subunit-like	Brachypodium distachyon	398
XP_002307042.2	hypothetical protein POPTR_0005s06690g	Populus trichocarpa	397
XP_007137783.1	hypothetical protein PHAVU_009G155200g	Phaseolus vulgaris	396
XP_006339225.1	PREDICTED: DNA polymerase delta catalytic subunit-like	Solanum tuberosum	396
NP_001238693.1	DNA polymerase delta catalytic subunit	Glycine max	396
XP_001641357.1	predicted protein	Nematostella vectensis	396
XP_005807050.1	PREDICTED: DNA polymerase delta catalytic subunit-like	Xiphophorus maculatus	395
XP_757605.1	hypothetical protein UM01458.1	Ustilago maydis 521	394
NP_001067405.1	Os11g0186400	Oryza sativa Japonica Group	394
NP_201201.2	DNA polymerase delta subunit 1	Arabidopsis thaliana	394
XP_004249349.1	PREDICTED: DNA polymerase delta catalytic subunit-like	Solanum lycopersicum	393
XP_004289285.1	PREDICTED: DNA polymerase delta catalytic subunit-like	Fragaria vesca subsp. vesca	392
XP_006663265.1	PREDICTED: DNA polymerase delta catalytic subunit-like	Oryza brachyantha	392
XP_004978893.1	PREDICTED: DNA polymerase delta catalytic subunit-like	Setaria italica	391
XP_007423155.1	PREDICTED: DNA polymerase delta catalytic subunit isoform	Python bivittatus	391
NP_035261.3	DNA polymerase delta catalytic subunit	Mus musculus	391
XP_006847158.1	hypothetical protein AMTR_s00017p00241740	Amborella trichopoda	390

XP_004071560.1	PREDICTED: DNA polymerase delta catalytic subunit-like	Oryzias latipes	390
XP_003222764.2	PREDICTED: DNA polymerase delta catalytic subunit	Anolis carolinensis	389
XP_007148845.1	hypothetical protein PHAVU_005G019000g	Phaseolus vulgaris	388
XP_002864879.1	DNA-directed DNA polymerase delta catalytic subunit	Arabidopsis lyrata subsp. lyrata	388
XP_002451581.1	hypothetical protein SORBIDRAFT_04g004200	Sorghum bicolor	388
XP_007910222.1	PREDICTED: DNA polymerase delta catalytic subunit	Callorhinchus milii	388
XP_002450450.1	hypothetical protein SORBIDRAFT_05g005680	Sorghum bicolor	388
XP_006465085.1	PREDICTED: DNA polymerase delta catalytic subunit-like isofo	Citrus sinensis	387
XP_006432146.1	hypothetical protein CICLE_v10000086mg	Citrus clementina	387
XP_007386579.1	delta DNA polymerase	Punctularia strigosozonata HHB-11173 SS5	386
XP_002264385.1	PREDICTED: DNA polymerase delta catalytic subunit	Vitis vinifera	387
XP_955596.1	DNA polymerase delta catalytic subunit	Encephalitozoon cuniculi GB-M1	384
XP_004866882.1	PREDICTED: DNA polymerase delta catalytic subunit isoform	Heterocephalus glaber	387
XP_004866881.1	PREDICTED: DNA polymerase delta catalytic subunit isoform	Heterocephalus glaber	387
XP_003073533.1	DNA polymerase catalytic subunit delta	Encephalitozoon intestinalis ATCC 50506	383
XP_005341718.1	PREDICTED: DNA polymerase delta catalytic subunit isoform	Ictidomys tridecemlineatus	386
NP_067694.1	DNA polymerase delta catalytic subunit	Rattus norvegicus	385
XP_006229204.1	PREDICTED: DNA polymerase delta catalytic subunit isoform	Rattus norvegicus	385
NP_001167152.1	DNA polymerase delta catalytic subunit	Salmo salar	385
NP_001039204.1	DNA polymerase delta catalytic subunit	Xenopus (Silurana) tropicalis	385
XP_003465533.1	PREDICTED: DNA polymerase delta catalytic subunit	Cavia porcellus	385
XP_007531357.1	PREDICTED: DNA polymerase delta catalytic subunit	Erinaceus europaeus	385
XP_005366922.1	PREDICTED: DNA polymerase delta catalytic subunit	Microtus ochrogaster	384
XP_005412847.1	PREDICTED: DNA polymerase delta catalytic subunit	Chinchilla lanigera	384
NP_776852.1	DNA polymerase delta catalytic subunit	Bos taurus	384
XP_001762435.1	predicted protein	Physcomitrella patens	385
XP_007179991.1	PREDICTED: DNA polymerase delta catalytic subunit isoform	Balaenoptera acutorostrata scammoni	384
NP_001190611.1	DNA polymerase delta subunit 1	Arabidopsis thaliana	384
XP_004646468.1	PREDICTED: DNA polymerase delta catalytic subunit	Octodon degus	383
XP_003801504.1	PREDICTED: DNA polymerase delta catalytic subunit	Otolemur garnettii	383
NP_001087694.1	DNA-directed DNA polymerase delta 1	Xenopus laevis	382
XP_007463489.1	PREDICTED: DNA polymerase delta catalytic subunit	Lipotes vexillifer	382
XP_851285.1	PREDICTED: DNA polymerase delta catalytic subunit isoform)	Canis lupus familiaris	382
XP_003916004.1	PREDICTED: DNA polymerase delta catalytic subunit	Papio anubis	382
XP_004440369.1	PREDICTED: DNA polymerase delta catalytic subunit	Ceratotherium simum simum	382
XP_005180680.1	PREDICTED: DNA polymerase delta catalytic subunit-like	Musca domestica	380
XP_001353021.1	GA19253	Drosophila pseudoobscura pseudoobscura	381
XP_006905068.1	PREDICTED: DNA polymerase delta catalytic subunit	Pteropus alecto	382
XP_004286191.1	PREDICTED: DNA polymerase delta catalytic subunit isoform	Orcinus orca	381
XP_005706542.1	DNA polymerase delta subunit 1	Galdieria sulphuraria	380
XP_005590064.1	PREDICTED: DNA polymerase delta catalytic subunit isoform	Macaca fascicularis	381
XP_004326518.1	PREDICTED: DNA polymerase delta catalytic subunit	Tursiops truncatus	381
XP_002554637.1	KLTH0F09966p	Lachancea thermotolerans CBS 6340	380
XP_007362961.1	hypothetical protein DICSQDRAFT_81194	Dichomitus squalens LYAD-421 SS1	380
XP_003964532.1	PREDICTED: DNA polymerase delta catalytic subunit-like	Takifugu rubripes	380
XP_006986263.1	PREDICTED: DNA polymerase delta catalytic subunit	Peromyscus maniculatus bairdii	380
XP_006208466.1	PREDICTED: LOW QUALITY PROTEIN: DNA polymerase delta c	Vicugna pacos	380
XP_006274455.1	PREDICTED: DNA polymerase delta catalytic subunit	Alligator mississippiensis	380

XP_006015096.1	PREDICTED: DNA polymerase delta catalytic subunit	Alligator sinensis	380
XP_001493912.1	PREDICTED: DNA polymerase delta catalytic subunit	Equus caballus	380
XP_002399458.1	DNA polymerase delta catalytic subunit, putative	Ixodes scapularis	378
XP_002568889.1	Pc21g18980	Penicillium chrysogenum Wisconsin 54-1255	380
XP_004209898.1	PREDICTED: DNA polymerase delta catalytic subunit-like	Hydra vulgaris	379
XP_001883646.1	predicted protein	Laccaria bicolor S238N-H82	379
NP_002682.2	DNA polymerase delta catalytic subunit	Homo sapiens	379
XP_005084828.1	PREDICTED: DNA polymerase delta catalytic subunit	Mesocricetus auratus	379
XP_004672610.1	PREDICTED: DNA polymerase delta catalytic subunit isoform	Jaculus jaculus	379
XP_006868213.1	PREDICTED: DNA polymerase delta catalytic subunit isoform	Chrysochloris asiatica	379
XP_001973157.1	GG13508	Drosophila erecta	378
XP_002086418.1	GE23127	Drosophila yakuba	378
NP_524099.2	DNA-polymerase-delta	Drosophila melanogaster	378
XP_007304064.1	hypothetical protein STEHIDRAFT_79581	Stereum hirsutum FP-91666 SS1	377
XP_003887919.1	DNA polymerase type-B delta catalytic subunit	Encephalitozoon hellem ATCC 50504	375
XP_001116065.1	PREDICTED: DNA polymerase delta catalytic subunit isoform	Macaca mulatta	382
XP_002995919.1	hypothetical protein NCER_101069	Nosema ceranae BRL01	375
XP_002762441.1	PREDICTED: DNA polymerase delta catalytic subunit	Callithrix jacchus	378
XP_001957325.1	GF10365	Drosophila ananassae	377
XP_007865168.1	hypothetical protein GLOTRDRAFT_120926	Gloeophyllum trabeum ATCC 11539	377
XP_004409979.1	PREDICTED: DNA polymerase delta catalytic subunit isoform	Odobenus rosmarus divergens	377
XP_003813667.1	PREDICTED: DNA polymerase delta catalytic subunit	Pan paniscus	377
XP_002983728.1	hypothetical protein SELMODRAFT_180322	Selaginella moellendorffii	375
XP_006282068.1	hypothetical protein CARUB_v10028313mg	Capsella rubella	377
XP_001602680.2	PREDICTED: DNA polymerase delta catalytic subunit-like	Nasonia vitripennis	377
XP_002085037.1	GD12524	Drosophila simulans	377
XP_001892851.1	DNA polymerase delta catalytic subunit	Brugia malayi	376
XP_004619921.1	PREDICTED: DNA polymerase delta catalytic subunit	Sorex araneus	377
XP_007995836.1	PREDICTED: DNA polymerase delta catalytic subunit isoform	Chlorocebus sabaeus	377
XP_003127412.2	PREDICTED: DNA polymerase delta catalytic subunit isoform	Sus scrofa	376
XP_006749288.1	PREDICTED: DNA polymerase delta catalytic subunit	Leptonychotes weddellii	376
XP_005991045.1	PREDICTED: DNA polymerase delta catalytic subunit	Latimeria chalumnae	375
XP_002047697.1	GJ11779	Drosophila virilis	375
XP_007623475.1	PREDICTED: DNA polymerase delta catalytic subunit isoform	Cricetulus griseus	375
XP_002008314.1	GI13419	Drosophila mojavensis	375
XP_005590063.1	PREDICTED: DNA polymerase delta catalytic subunit isoform	Macaca fascicularis	375
XP_007400698.1	hypothetical protein PHACADRAFT_104509	Phanerochaete carnosa HHB-10118-sp	374
XP_008038269.1	delta DNA polymerase	Trametes versicolor FP-101664 SS1	374
XP_004381756.1	PREDICTED: DNA polymerase delta catalytic subunit	Trichechus manatus latirostris	375
XP_001948892.2	PREDICTED: DNA polymerase delta catalytic subunit-like	Acyrthosiphon pisum	374
XP_772922.1	hypothetical protein CNBK2930	Cryptococcus neoformans var. neoformans B-3501A	374
XP_003647178.1	hypothetical protein Ecym_5625	Eremothecium cymbalariae DBVPG#7215	374
XP_004767275.1	PREDICTED: DNA polymerase delta catalytic subunit isoform	Mustela putorius furo	374
XP_004030429.1	hypothetical protein IMG5_161090	Ichthyophthirius multifiliis	374
XP_454020.1	hypothetical protein	Kluyveromyces lactis NRRL Y-1140	374
XP_004597010.1	PREDICTED: DNA polymerase delta catalytic subunit	Ochotona princeps	374
XP_003725042.1	PREDICTED: DNA polymerase delta catalytic subunit-like	Strongylocentrotus purpuratus	380
XP_006941017.1	PREDICTED: DNA polymerase delta catalytic subunit	Felis catus	377

XP_003196775.1	delta DNA polymerase	Cryptococcus gattii WM276	372
XP_007995839.1	PREDICTED: DNA polymerase delta catalytic subunit isoform	Chlorocebus sabaeus	377
XP_004767274.1	PREDICTED: DNA polymerase delta catalytic subunit isoform	Mustela putorius furo	374
XP_001606357.1	PREDICTED: DNA polymerase delta catalytic subunit-like	Nasonia vitripennis	372
XP_004767273.1	PREDICTED: DNA polymerase delta catalytic subunit isoform	Mustela putorius furo	374
XP_006080226.1	PREDICTED: DNA polymerase delta catalytic subunit	Bubalus bubalis	375
XP_002069178.1	GK23632	Drosophila willistoni	371
XP_007770892.1	hypothetical protein CONPUDRAFT_138364	Coniophora puteana RWD-64-598 SS2	371
XP_004993090.1	polymerase	Salpingoeca rosetta	372
XP_007343979.1	hypothetical protein AURDEDRAFT_102277	Auricularia delicata TFB-10046 SS5	370
XP_004767271.1	PREDICTED: DNA polymerase delta catalytic subunit isoform	Mustela putorius furo	374
XP_004767270.1	PREDICTED: DNA polymerase delta catalytic subunit isoform	Mustela putorius furo	374
XP_001397242.1	DNA polymerase delta catalytic subunit	Aspergillus niger CBS 513.88	371
XP_808567.1	DNA polymerase delta catalytic subunit	Trypanosoma cruzi strain CL Brener	369
XP_007842982.1	dna polymerase delta catalytic subunit	Moniliophthora roreri MCA 2997	370
XP_001689909.1	DNA polymerase delta subunit one	Chlamydomonas reinhardtii	370
XP_007876498.1	hypothetical protein PFL1_00805	Pseudozyma flocculosa PF-1	369
XP_003878423.1	putative DNA polymerase delta catalytic subunit	Leishmania mexicana MHOM/GT/2001/U1103	369
XP_003033238.1	hypothetical protein SCHCODRAFT_81939	Schizophyllum commune H4-8	369
XP_001868453.1	DNA polymerase delta catalytic subunit	Culex quinquefasciatus	370
XP_005879691.1	PREDICTED: DNA polymerase delta catalytic subunit	Myotis brandtii	369
XP_003143198.1	DNA-directed DNA polymerase III	Loa loa	369
XP_007006270.1	hypothetical protein TREMEDRAFT_69477	Tremella mesenterica DSM 1558	368
XP_567620.1	delta DNA polymerase	Cryptococcus neoformans var. neoformans JEC21	368
XP_002917866.1	PREDICTED: LOW QUALITY PROTEIN: DNA polymerase delta c	Ailuropoda melanoleuca	369
XP_002982737.1	hypothetical protein SELMODRAFT_155333	Selaginella moellendorffii	366
XP_005259063.1	PREDICTED: DNA polymerase delta catalytic subunit isoform	Homo sapiens	369
XP_002125320.1	PREDICTED: DNA polymerase delta catalytic subunit-like	Ciona intestinalis	367
XP_003682859.1	hypothetical protein TDEL_0G02810	Torulaspora delbrueckii	368
XP_002429857.1	DNA polymerase delta catalytic subunit, putative	Pediculus humanus corporis	367
XP_001728836.1	hypothetical protein MGL_4003	Malassezia globosa CBS 7966	365
XP_002956681.1	hypothetical protein VOLCADRAFT_67281	Volvox carteri f. nagariensis	365
XP_006897331.1	PREDICTED: DNA polymerase delta catalytic subunit isoform	Elephantulus edwardii	367
XP_003484917.1	PREDICTED: DNA polymerase delta catalytic subunit-like isofo	Bombus impatiens	367
XP_005664840.1	PREDICTED: DNA polymerase delta catalytic subunit isoform	Sus scrofa	367
XP_951513.1	DNA polymerase delta catalytic subunit	Trypanosoma brucei brucei strain 927/4 GUTat10.1	365
XP_003484918.1	PREDICTED: DNA polymerase delta catalytic subunit-like isofo	Bombus impatiens	367
XP_007261467.1	hypothetical protein FOMMEDRAFT_164466	Fomitiporia mediterranea MF3/22	366
XP_001211449.1	DNA polymerase delta catalytic subunit	Aspergillus terreus NIH2624	367
XP_002174009.1	DNA polymerase delta catalytic subunit	Schizosaccharomyces japonicus yFS275	366
XP_007320677.1	hypothetical protein SERLADRAFT_451027	Serpula lacrymans var. lacrymans S7.9	365
XP_001685930.1	putative DNA polymerase delta catalytic subunit	Leishmania major strain Friedlin	365
XP_002494695.1	ZYRO0A07524p	Zygosaccharomyces rouxii CBS 732	366
NP_010181.2	Pol3p	Saccharomyces cerevisiae S288c	366
XP_003675047.1	hypothetical protein NCAS_0B05920	Naumovozyma castellii CBS 4309	366
XP_002486944.1	DNA polymerase delta catalytic subunit Cdc2, putative	Talaromyces stipitatus ATCC 10500	366
XP_002895592.1	DNA polymerase delta catalytic subunit	Phytophthora infestans T30-4	371
XP_001275938.1	DNA polymerase delta large chain	Aspergillus clavatus NRRL 1	365

XP_003864005.1	DNA polymerase delta catalytic subunit, putative	Leishmania donovani	363
XP_001468284.1	putative DNA polymerase delta catalytic subunit	Leishmania infantum JPCM5	363
XP_755997.1	DNA polymerase delta catalytic subunit Cdc2	Aspergillus fumigatus Af293	364
XP_001645504.1	hypothetical protein Kpol_1004p20	Vanderwaltozyma polyspora DSM 70294	364
XP_002550800.1	DNA polymerase delta catalytic subunit	Candida tropicalis MYA-3404	363
XP_006570048.1	PREDICTED: DNA polymerase delta catalytic subunit isoform	Apis mellifera	363
XP_003693840.1	PREDICTED: DNA polymerase delta catalytic subunit-like	Apis florea	363
XP_006614852.1	PREDICTED: DNA polymerase delta catalytic subunit-like isofo	Apis dorsata	362
XP_003284787.1	DNA polymerase delta catalytic subunit	Dictyostelium purpureum	362
NP_596124.1	DNA polymerase delta catalytic subunit Cdc6	Schizosaccharomyces pombe 972h-	363
XP_006570047.1	PREDICTED: DNA polymerase delta catalytic subunit isoform	Apis mellifera	363
XP_001822406.1	DNA polymerase delta catalytic subunit	Aspergillus oryzae RIB40	363
XP_623795.1	PREDICTED: DNA polymerase delta catalytic subunit isoform	Apis mellifera	363
XP_006462071.1	hypothetical protein AGABI2DRAFT_186127	Agaricus bisporus var. bisporus H97	362
XP_006959614.1	putative delta DNA polymerase	Wallemia sebi CBS 633.66	362
XP_003050401.1	predicted protein	Nectria haematococca mpVI 77-13-4	362
XP_006614851.1	PREDICTED: DNA polymerase delta catalytic subunit-like isofo	Apis dorsata	362
XP_004522162.1	PREDICTED: DNA polymerase delta catalytic subunit-like	Ceratitis capitata	362
XP_006614850.1	PREDICTED: DNA polymerase delta catalytic subunit-like isofo	Apis dorsata	362
XP_003668596.1	hypothetical protein NDAI_0B03180	Naumovozyma dairenensis CBS 421	362
XP_001261127.1	DNA polymerase delta large chain	Neosartorya fischeri NRRL 181	362
XP_003703291.1	PREDICTED: DNA polymerase delta catalytic subunit-like	Megachile rotundata	361
XP_001984922.1	GH16758	Drosophila grimshawi	360
XP_003685042.1	hypothetical protein TPHA_0C04580	Tetrapisispora phaffii CBS 4417	360
XP_002145577.1	DNA polymerase delta catalytic subunit Cdc2, putative	Talaromyces marneffei ATCC 18224	360
XP_003737481.1	PREDICTED: DNA polymerase delta catalytic subunit-like	Metaseiulus occidentalis	360
XP_003008371.1	DNA polymerase delta catalytic subunit	Verticillium alfalfae VaMs.102	359
XP_002421460.1	DNA polymerase III, putative; DNA polymerase delta catalytic	Candida dubliniensis CD36	358
XP_007915677.1	putative dna polymerase delta catalytic subunit protein	Togninia minima UCRPA7	360
XP_003396007.1	PREDICTED: DNA polymerase delta catalytic subunit-like isofo	Bombus terrestris	359
XP_001032353.1	DNA polymerase family B containing protein	Tetrahymena thermophila	360
XP_387351.1	hypothetical protein FG07175.1	Fusarium graminearum PH-1	359
XP_003396008.1	PREDICTED: DNA polymerase delta catalytic subunit-like isofo	Bombus terrestris	359
XP_006693331.1	DNA polymerase-like protein	Chaetomium thermophilum var. thermophilum DSM	359
XP_007327213.1	hypothetical protein AGABI1DRAFT_55049	Agaricus bisporus var. burnettii JB137-S8	358
XP_003715024.1	DNA polymerase delta catalytic subunit	Magnaporthe oryzae 70-15	358
XP_888889.1	hypothetical protein CaO19_5182	Candida albicans SC5314	357
XP_003855627.1	DNA polymerase delta catalytic subunit	Zymoseptoria tritici IPO323	358
XP_003056969.1	predicted protein	Micromonas pusilla CCMP1545	357
XP_002261685.1	dna polymerase delta catalytic subunit	Plasmodium knowlesi strain H	357
XP_001567964.1	putative DNA polymerase delta catalytic subunit	Leishmania braziliensis MHOM/BR/75/M2904	356
XP_001523599.1	DNA polymerase delta catalytic subunit	Lodderomyces elongisporus NRRL YB-4239	357
XP_961558.1	DNA polymerase delta catalytic subunit	Neurospora crassa OR74A	357
XP_002791431.1	DNA polymerase delta catalytic subunit	Paracoccidioides sp. 'lutzii' Pb01	356
XP_007357801.1	putative delta DNA polymerase	Auricularia delicata TFB-10046 SS5	353
XP_005838630.1	DNA polymerase delta catalytic subunit	Guillardia theta CCMP2712	353
XP_003871049.1	Pol3 large subunit of DNA polymerase III	Candida orthopsilosis Co 90-125	356
XP_003510946.1	PREDICTED: DNA polymerase delta catalytic subunit isoform	Cricetulus griseus	354

NP_985361.2	AFL189Wp	Ashbya gossypii ATCC 10895	354
XP_001648163.1	DNA polymerase delta catalytic subunit	Aedes aegypti	352
XP_499875.1	YALI0A08426p	Yarrowia lipolytica CLIB122	353
XP_001347450.1	DNA polymerase delta catalytic subunit	Plasmodium falciparum 3D7	354
XP_449097.1	hypothetical protein	Candida glabrata CBS 138	354
XP_729844.1	DNA polymerase delta catalytic subunit	Plasmodium yoelii yoelii 17XNL	354
XP_006394219.1	hypothetical protein EUTSA_v10003565mg	Eutrema salsugineum	353
XP_002770696.1	DEHA2G22308p	Debaryomyces hansenii CBS767	353
XP_007283296.1	DNA polymerase delta catalytic subunit	Colletotrichum gloeosporioides Nara gc5	354
XP_003269738.1	PREDICTED: LOW QUALITY PROTEIN: DNA polymerase delta c	Nomascus leucogenys	353
XP_005905083.1	PREDICTED: DNA polymerase delta catalytic subunit	Bos mutus	353
XP_005850494.1	DNA polymerase delta subunit	Chlorella variabilis	352
XP_006666602.1	DNA polymerase delta catalytic subunit	Cordyceps militaris CM01	353
XP_007412471.1	hypothetical protein MELLADRAFT_53062	Melampsora larici-populina 98AG31	352
XP_005259065.1	PREDICTED: DNA polymerase delta catalytic subunit isoform	Homo sapiens	352
XP_003959737.1	hypothetical protein KAFR_0K02460	Kazachstania africana CBS 2517	353
XP_003661712.1	hypothetical protein MYCTH_2301456	Myceliophthora thermophila ATCC 42464	352
XP_003842135.1	similar to catalytic subunit DNA polymerase delta	Leptosphaeria maculans JN3	352
XP_766420.1	DNA polymerase delta catalytic subunit	Theileria parva strain Muguga	352
XP_005537335.1	DNA polymerase delta, catalytic subunit	Cyanidioschyzon merolae strain 10D	352
XP_007788182.1	putative dna polymerase delta catalytic subunit protein	Eutypa lata UCREL1	351
XP_007674366.1	hypothetical protein BAUCODRAFT_31911	Baudoinia compniacensis UAMH 10762	352
XP_004365086.1	DNA polymerase delta1 catalytic subunit	Capsaspora owczarzaki ATCC 30864	355
XP_006960954.1	DNA polymerase delta, catalytic subunit	Trichoderma reesei QM6a	351
XP_967290.2	PREDICTED: similar to DNA polymerase delta catalytic subuni	Tribolium castaneum	352
XP_004367927.1	polymerase (DNA directed), delta 1, catalytic subunit	Acanthamoeba castellanii str. Neff	349
XP_001239851.1	hypothetical protein CIMG_09472	Coccidioides immitis RS	351
XP_004221328.1	DNA polymerase delta catalytic subunit	Plasmodium cynomolgi strain B	352
XP_002491049.1	Catalytic subunit of DNA polymerase delta	Komagataella pastoris GS115	350
XP_004179069.1	hypothetical protein TBLA_0B07320	Tetrapisispora blattae CBS 6284	350
XP_007601102.1	DNA polymerase family B	Colletotrichum fioriniae PJ7	350
XP_006432145.1	hypothetical protein CICLE_v10000086mg	Citrus clementina	348
YP_002959821.1	DNA-directed DNA polymerase, B family (pol)	Thermococcus gammatolerans EJ3	342
XP_003075187.1	DPOD1_ORYSA DNA polymerase delta catalytic subunit gb|A	Ostreococcus tauri	349
XP_003067381.1	DNA polymerase delta catalytic subunit, putative	Coccidioides posadasii C735 delta SOWgp	348
XP_006144719.1	PREDICTED: DNA polymerase delta catalytic subunit	Tupaia chinensis	348
XP_002502470.1	predicted protein	Micromonas sp. RCC299	349
XP_002626600.1	DNA polymerase delta subunit	Ajellomyces dermatitidis SLH14081	348
YP_004762632.1	DNA-directed DNA polymerase, B family (pol)	Thermococcus sp. 4557	341
XP_004200106.1	Piso0_002676	Millerozyma farinosa CBS 7064	347
XP_007377768.1	DNA polymerase delta catalytic subunit	Spathaspora passalidarum NRRL Y-27907	347
XP_001225371.1	conserved hypothetical protein	Chaetomium globosum CBS 148.51	348
XP_001326973.1	polymerase zeta subunit	Trichomonas vaginalis G3	347
XP_007923896.1	hypothetical protein MYCFIDRAFT_202624	Pseudocercospora fijiensis CIRAD86	347
XP_007834989.1	DNA polymerase delta catalytic subunit	Pestalotiopsis fici W106-1	347
XP_002108586.1	hypothetical protein TRIADDRAFT_18979	Trichoplax adhaerens	346
XP_005646695.1	DNA polymerase delta subunit one	Coccomyxa subellipsoidea C-169	347
XP_001831621.1	delta DNA polymerase	Coprinopsis cinerea okayama7#130	347

XP_001612703.1	DNA polymerase delta catalytic subunit	Plasmodium vivax Sal-1	346
XP_001486690.1	hypothetical protein PGUG_00067	Meyerozyma guilliermondii ATCC 6260	346
XP_006678898.1	hypothetical protein BATDEDRAFT_16581	Batrachochytrium dendrobatidis JAM81	347
XP_638283.1	DNA polymerase delta catalytic subunit	Dictyostelium discoideum AX4	346
XP_002181135.1	DNA polymerase delta	Phaeodactylum tricornutum CCAP 1055/1	346
XP_007213679.1	hypothetical protein PRUPE_ppa000929mg	Prunus persica	343
XP_003330341.2	DNA polymerase delta subunit 1	Puccinia graminis f. sp. tritici CRL 75-36-700-3	345
XP_001940287.1	DNA polymerase delta catalytic subunit	Pyrenophora tritici-repentis Pt-1C-BFP	345
XP_003344540.1	hypothetical protein SMAC_07548	Sordaria macrospora k-hell	344
XP_007814725.1	DNA polymerase delta catalytic subunit	Metarhizium acridum CQMa 102	345
XP_007749672.1	DNA polymerase delta subunit 1	Cladophialophora psammophila CBS 110553	345
YP_004423780.1	DNA polymerase I	Pyrococcus sp. NA2	337
XP_003649231.1	hypothetical protein THITE_2107682	Thielavia terrestris NRRL 8126	344
XP_003299297.1	hypothetical protein PTT_10256	Pyrenophora teres f. teres 0-1	344
XP_007802018.1	DNA polymerase delta catalytic subunit	Endocarpon pusillum Z07020	344
XP_007733170.1	DNA polymerase delta subunit 1	Capronia epimyces CBS 606.96	344
XP_007825648.1	DNA polymerase delta catalytic subunit	Metarhizium anisopliae ARSEF 23	343
XP_007761251.1	DNA polymerase delta subunit 1	Cladophialophora yegresii CBS 114405	343
XP_680594.1	hypothetical protein AN7325.2	Aspergillus nidulans FGSC A4	342
XP_003023378.1	Catalytic subunit of DNA polymerase delta	Trichophyton verrucosum HKI 0517	343
XP_007586663.1	putative dna polymerase delta catalytic subunit protein	Neofusicoccum parvum UCRNP2	343
XP_001386065.2	DNA polymerase delta catalytic subunit (DNA polymerase III)	Scheffersomyces stipitis CBS 6054	342
XP_004199250.1	Piso0_002676	Millerozyma farinosa CBS 7064	342
XP_953875.1	DNA polymerase delta catalytic subunit (Pold1)	Theileria annulata strain Ankara	342
XP_001827844.1	DNA polymerase delta catalytic subunit	Enterocytozoon bieneusi H348	340
XP_008022340.1	hypothetical protein SETTUDRAFT_175540	Setosphaeria turcica Et28A	343
XP_006723311.1	PREDICTED: DNA polymerase delta catalytic subunit isoform	Homo sapiens	342
XP_007728200.1	DNA polymerase delta subunit 1	Capronia coronata CBS 617.96	342
XP_003234720.1	DNA polymerase subunit delta large	Trichophyton rubrum CBS 118892	342
XP_003010549.1	Catalytic subunit of DNA polymerase delta	Arthroderma benhamiae CBS 112371	341
XP_001416112.1	predicted protein	Ostreococcus lucimarinus CCE9901	338
XP_002848356.1	DNA polymerase delta catalytic subunit	Arthroderma otae CBS 113480	340
XP_003170543.1	DNA polymerase delta catalytic subunit	Arthroderma gypseum CBS 118893	340
XP_006685056.1	DNA polymerase delta catalytic subunit	Candida tenuis ATCC 10573	338
XP_001587512.1	hypothetical protein SS1G_11504	Sclerotinia sclerotiorum 1980 UF-70	339
XP_006763939.1	PREDICTED: DNA polymerase delta catalytic subunit	Myotis davidii	335
XP_007705446.1	hypothetical protein COCSADRAFT_153806	Bipolaris sorokiniana ND90Pr	339
XP_001801013.1	hypothetical protein SNOG_10753	Phaeosphaeria nodorum SN15	339
XP_002291074.1	DNA polymerase	Thalassiosira pseudonana CCMP1335	337
NP_577941.1	DNA-directed DNA polymerase	Pyrococcus furiosus DSM 3638	330
XP_005710748.1	unnamed protein product	Chondrus crispus	334
NP_127396.1	DNA polymerase I	Pyrococcus abyssi GE5	328
XP_007179992.1	PREDICTED: DNA polymerase delta catalytic subunit isoform	Balaenoptera acutorostrata scammoni	333
XP_004350202.1	DNA polymerase delta catalytic subunit	Dictyostelium fasciculatum	340
XP_005616326.1	PREDICTED: DNA polymerase delta catalytic subunit isoform	Canis lupus familiaris	331
XP_002608243.1	hypothetical protein BRAFLDRAFT_125058	Branchiostoma floridae	336
XP_004286192.1	PREDICTED: DNA polymerase delta catalytic subunit isoform	Orcinus orca	331
XP_007294227.1	DNA polymerase family B	Marssonina brunnea f. sp. 'multigermtubi' MB_m1	332

XP_004833491.1	DNA polymerase delta catalytic subunit, putative	Babesia equi	330
XP_001610797.1	DNA polymerase delta subunit	Babesia bovis T2Bo	330
XP_008078743.1	DNA/RNA polymerase	Glarea lozoyensis ATCC 20868	330
XP_004409980.1	PREDICTED: DNA polymerase delta catalytic subunit isoform	Odobenus rosmarus divergens	327
XP_003378671.1	DNA polymerase delta catalytic subunit	Trichinella spiralis	329
XP_002636649.1	Hypothetical protein CBG23360	Caenorhabditis briggsae	329
NP_506017.1	Protein F10C2.4	Caenorhabditis elegans	328
XP_007514118.1	DNA polymerase delta catalytic subunit	Bathycoccus prasinos	328
XP_805939.1	DNA polymerase delta catalytic subunit	Trypanosoma cruzi strain CL Brener	321
XP_627772.1	DNA polymerase delta catalytic subunit. DNAQ-like 3'-5' exor	Cryptosporidium parvum Iowa II	325
NP_614322.1	B family DNA polymerase	Methanopyrus kandleri AV19	320
YP_004483758.1	DNA polymerase Pol2	Methanotorris igneus Kol 5	319
YP_008604717.1	DNA polymerase II	Aeropyrum camini SY1 = JCM 12091	318
XP_006191496.1	PREDICTED: DNA polymerase delta catalytic subunit	Camelus ferus	322
XP_002839850.1	hypothetical protein	Tuber melanosporum Mel28	323
YP_003356358.1	family B DNA polymerase	Methanocella paludicola SANAE	315
XP_005433882.1	PREDICTED: LOW QUALITY PROTEIN: DNA polymerase delta (	Falco cherrug	318
XP_003114454.1	hypothetical protein CRE_27041	Caenorhabditis remanei	318
NP_378066.1	DNA polymerase II	Sulfolobus tokodaii str. 7	312
XP_001443230.1	hypothetical protein	Paramecium tetraurelia strain d4-2	316
YP_005380898.1	replicative DNA polymerase I	Methanocella conradii HZ254	312
XP_001748370.1	hypothetical protein	Monosiga brevicollis MX1	319
YP_003435908.1	DNA polymerase Pol2	Ferroglobus placidus DSM 10642	311
XP_007955835.1	PREDICTED: DNA polymerase delta catalytic subunit	Orycteropus afer afer	311
YP_003400016.1	DNA polymerase Pol2	Archaeoglobus profundus DSM 5631	308
XP_002142640.1	DNA polymerase family B	Cryptosporidium muris RN66	313
NP_148383.2	DNA polymerase II	Aeropyrum pernix K1	307
YP_007906237.1	replicative DNA polymerase I	Archaeoglobus sulfaticallidus PM70-1	306
YP_687422.1	DNA-directed DNA polymerase B	Methanocella arvoryzae MRE50	305
YP_007247297.1	DNA polymerase elongation subunit (family B)	Aciduliprofundum sp. MAR08-339	304
WP_023429130.1	DNA polymerase (pol2)	uncultured Acidilobus sp. JCHS	303
YP_007174945.1	DNA polymerase pol2	Caldisphaera lagunensis DSM 15908	301
YP_004342252.1	DNA polymerase Pol2	Archaeoglobus veneficus SNP6	301
YP_003816790.1	DNA polymerase 2	Acidilobus saccharovorans 345-15	300
